# Longitudinal metabolomics of human plasma reveal metabolic dynamics and predictive markers of antituberculosis drug-induced liver injury

**DOI:** 10.1186/s12931-024-02837-8

**Published:** 2024-06-21

**Authors:** Mengjiao Li, Dan Zhang, Qingxin Yang, Zhenzhen Zhao, Chunying Zhang, Yanbing Zhou, Yangjuan Bai, Lu Chen, Xiaoyan Tang, Cuihua Liu, Juan Zhou, Xuerong Chen, Binwu Ying

**Affiliations:** 1 Department of Laboratory Medicine, West China Hospital, Sichuan University, Chengdu, China; 2 Department of Laboratory Medicine, State Key Laboratory of Biotherapy and Collaborative Innovation Center for Biotherapy, West China Hospital, Sichuan University, Chengdu, China; 3 Department of Respiratory and Critical Care Medicine, West China Hospital, Sichuan University, Chengdu, China; 4 CAS Key Laboratory of Pathogenic Microbiology and Immunology, Institute of Microbiology, Chinese Academy of Sciences, Beijing, China

**Keywords:** Tuberculosis, Drug-induced liver injury, Metabolomics, Biomarker

## Abstract

Tuberculosis (TB) remains the second leading cause of death from a single infectious agent and long-term medication could lead to antituberculosis drug-induced liver injury (ATB-DILI). We established a prospective longitudinal cohort of ATB-DILI with multiple timepoint blood sampling and used untargeted metabolomics to analyze the metabolic profiles of 107 plasma samples from healthy controls and newly diagnosed TB patients who either developed ATB-DILI within 2 months of anti-TB treatment (ATB-DILI subjects) or completed their treatment without any adverse drug reaction (ATB-Ctrl subjects). The untargeted metabolome revealed that 77 metabolites (of 895 total) were significantly changed with ATB-DILI progression. Among them, levels of multiple fatty acids and bile acids significantly increased over time in ATB-DILI subjects. Meanwhile, metabolites of the same class were highly correlated with each other and pathway analysis indicated both fatty acids metabolism and bile acids metabolism were up-regulated with ATB-DILI progression. The targeted metabolome further validated that 5 fatty acids had prediction capability at the early stage of the disease and 6 bile acids had a better diagnostic performance when ATB-DILI occurred. These findings provide evidence indicating that fatty acids metabolism and bile acids metabolism play a vital role during ATB-DILI progression. Our report adds a dynamic perspective better to understand the pathological process of ATB-DILI in clinical settings.

**Supplementary Information:**

The online version contains supplementary material available at 10.1186/s12931-024-02837-8.

## Introduction

Tuberculosis (TB) remains a formidable global health challenge, ranking as the second leading cause of death among infectious diseases worldwide, second only to COVID-19 [1]. Treatment for newly diagnosed TB patients involves a standard regimen consisting of an intensive phase of four drugs (isoniazid, rifampicin, pyrazinamide, and ethambutol) for two months, followed by a continuation phase of two drugs (isoniazid and rifampicin) for four months [2]. However, long-term medication can result in various adverse drug reactions, leading to the modification or interruption of treatment, developing drug resistance, and even treatment failure.

One of the most common adverse reactions is antituberculosis drug-induced liver injury (ATB-DILI), which can vary from the asymptomatic elevation of alanine aminotransferase (ALT) to acute hepatic failure, with an incidence rate of 0.8–40% [3]. Patients who developed ATB-DILI were at a higher risk of prolonged intensive treatment and unsuccessful treatment outcomes [4]. Therefore, ATB-DILI remains a severe clinical problem that requires prompt detection and proper management.

Although recent studies have highlighted the impact of genetic polymorphisms (*NAT2*, *GSTT*, *GSTM*, *CYP2E1*, *HLA-DQA1/DQB1*) on the risk of ATB-DILI, these variations account for only a fraction of the cases because gene transcription and expression could be affected by environmental factors and immune status [3, 5]. Therefore, it is crucial to explore novel biomarkers beyond genetic polymorphism to gain a comprehensive understanding of the disease.

Previous studies have shown that isoniazid and its metabolites changed hepatic steatosis-associated gene expression, including lipid synthesis, transport, and metabolism genes [6]. Comparative toxicoproteomics studies illustrated that proteins related to the peroxisome proliferator-activated receptor-γ (PPARγ) signaling pathway, cytochrome P450, and glutathione metabolism were up-regulated while proteins related to arachidonic acid metabolism were down-regulated [7]. All the above research indicated that gene transcription and protein expression of lipid metabolism might be associated with ATB-DILI. However, metabolite changes in lipid metabolism during ATB-DILI remain to be explored.

The metabolites, as the downstream products of genomic, transcriptomic, and proteomic, are the crucial precursors and substrates involved in energy supply, signal transduction, and cell proliferation. Disrupted metabolic homeostasis is supposed to contribute to disease onset and progression [8]. Therefore, metabolites have the potential to serve as biomarkers for predicting disease occurrence and progression.

Recent metabolomic studies reported low serum or cerebrospinal fluid tryptophan concentrations were strongly associated with the progression of pulmonary tuberculosis or tuberculosis meningitis [9, 10]. Animal studies further confirmed indoleamine 2,3-dioxygenase (IDO) catabolizes tryptophan to downstream metabolites and suppression of IDO activity reduces bacterial burden, alleviates clinical symptoms, and increases host survival [11]. Increasing research also suggested that host lipids like cholesteryl esters, oxylipin, ceramides, triglycerides, and phosphatidylcholine showed a clear expression pattern during antituberculosis treatment, which could be potential biomarkers for therapeutic monitoring [12, 13]. These studies provided a classic example for metabolomics application in ATB-DILI. Recent studies have reported that individuals with ATB-DILI exhibit distinctive metabolic profiles in their plasma and urine samples [14, 15]. However, cross-sectional data have limited predictive ability and do not provide insights into how metabolite levels change with the progression of ATB-DILI.

Here, we conducted a study aiming to find prediction biomarkers for ATB-DILI using both untargeted and targeted metabolomics approaches in longitudinal and cross-sectional cohorts. By comprehensively analyzing metabolite profiles and their longitudinal dynamics, we sought to identify prediction biomarkers that could aid in the early detection, management, and prognosis of ATB-DILI.

## Methods

### Establishment of clinical cohort

**Longitudinal cohort**: We enrolled newly diagnosed TB patients at West China Hospital, Sichuan University. These patients received a standard treatment regimen comprising rifampin (RIF, 450 mg/day) or rifapentine (RFT, 600 mg/twice a week), isoniazid (INH, 300 mg/day), ethambutol (EMB, 750 mg/day), and pyrazinamide (PZA, 1500 mg/day) during the intensive phase, followed by RIF or RFT, INH, and EMB during the continuation phase. At enrollment, all patients were confirmed by experienced clinicians, based on clinical symptoms, microbiological or radiological evidence [16]. The exclusion criteria were as follows: diabetes mellitus, chronic liver diseases, chronic renal diseases, malignant tumor, autoimmune diseases, human immunodeficiency virus infection, or long-term use of traditional medicine.

Since the start of antituberculosis treatment, all participants underwent weekly visits in the first month (1 W, 2 W, 3 W, 4 W) and biweekly visits in the second month (6 W, 8 W). According to whether develops ATB-DILI, patients were divided into ATB-DILI group and antituberculosis control (ATB-Ctrl) group. All ATB-DILI subjects were confirmed based on the following criteria: (1) ALT ≥ 3×upper limit of normal (ULN) with hepatitis symptoms such as nausea, vomiting, and jaundice, or (2) ALT ≥ 5×ULN with or without clinical symptoms, or (3) aspartate aminotransferase (AST), alkaline phosphatase (ALP), and total bilirubin (TBIL) elevated simultaneously and at least one ≥ 2×ULN [17, 18].

#### Cross-sectional cohort

A total of 67 cases with ATB-DILI and 61 controls without any adverse events were enrolled in the cross-sectional cohort from October 2020 to December 2020, that were cross-sectional drug-induced liver injury (CS-DILI) group and cross-sectional control (CS-Ctrl) group. The exclusion criteria were the same as the longitudinal cohort. Plasma samples were collected within 7 days after ATB-DILI onset.

#### Healthy control cohort

A total of 50 healthy controls were randomly recruited from Physical Examination Center of West China Hospital of Sichuan University from January 2021 to June 2021.

#### Ethics approval and consent to participate

This study was approved by the Ethnics Committee of West China Hospital of Sichuan University (reference no. 829, 2019) following the World Medical Association’s Declaration of Helsinki. Written informed content was obtained from patients involved in the cohort.

### Metabolomic profiling

#### Untargeted metabolomics analysis

The EDTA anticoagulated plasma samples were collected from all participants for metabolomic analysis at Discovery HD4™ Platform (supplementary methods) [19].

#### Targeted metabolomics analysis

Quantitation of 5 fatty acids (FAs) and 6 bile acids (BAs) were performed with ultra performance liquid chromatography/tandem mass spectrometry (UPLC-MS/MS) (supplementary methods, supplementary Tables 1, 2, 3, 4, 5, 6).

### Statistical analysis

Data analysis was performed using SPSS 20.0 or GraphPad Prism 8.0 or R (version 4.1.0). Two-way repeated ANOVA was performed for repeated measurement data at multiple timepoints of the longitudinal cohort and adjusted *p* values were calculated using False Discovery Rate (FDR). Volcano plot, principal component analysis (PCA), partial least squares discriminant analysis (PLS-DA), weighted co-expression network analysis, dynamic feature cluster analysis and random forest classification model were performed by using R packages (ggplot2, mixOmics, WGCNA, Mfuzz, and Random Forest). Enrichment and pathway analysis of different metabolites were performed on the web of MetaboAnalyst 5.0 (https://www.metaboanalyst.ca). Multiple group comparisons of cross-sectional cohorts were performed by Kruskal Wallis test.

## Results

### ATB-DILI longitudinal cohort: a prospective study with high-density sampling

We enrolled 118 newly diagnosed TB patients and established a longitudinal cohort with multiple timepoints blood sampling (baseline and 1 W, 2 W, 3 W, 4 W, 6 W, 8 W after medication). A total of 14 individuals who developed DILI were included in ATB-DILI group and age-, gender-, and body mass index (BMI)-matched 14 individuals who complete their treatment without any adverse drug effects were included in ATB-Ctrl group.

**Fig. 1:**
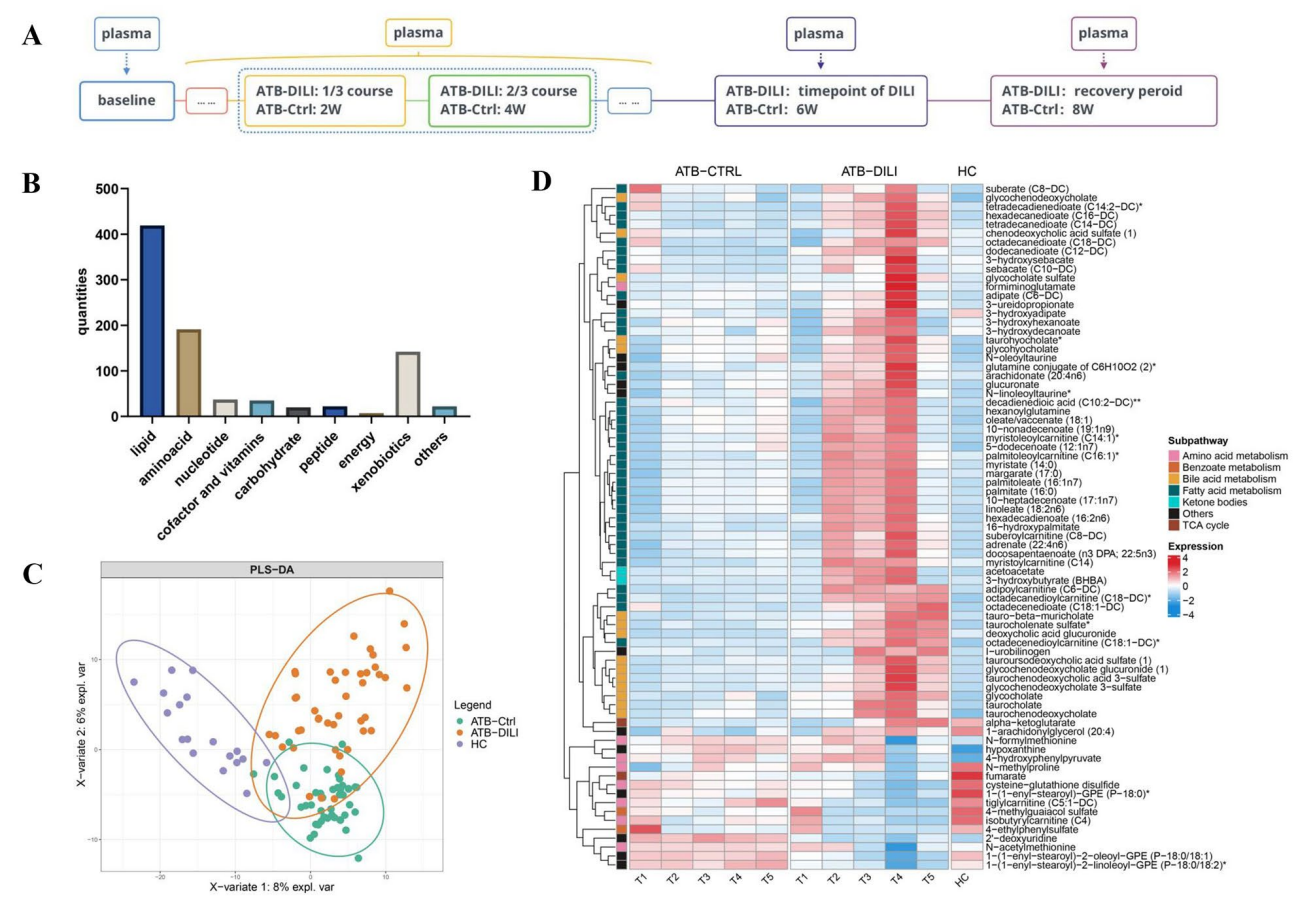
Untargeted metabolomics reveals dynamics changes of ATB-DILI. **(A)**: The ATB-DILI group: T1 (baseline), T2 (one-third course), T3 (two-third course), T4 (ATB-DILI), and T5 (recovery period). The ATB-Ctrl group: T1 (baseline), T2 (2 W), T3 (4 W), T4 (6 W), and T5 (8 W). **(B)** Metabolites compositions of global metabolomics. **(C)** Sample distribution of PLS-DA. **(D)** Heatmap displayed the average intensity of top77 metabolites

Demographics and clinical baseline characteristics are presented in Table 1 and therapeutic regimes for each patient are listed in the supplementary Table 7. Dynamic changes of ALT and AST were displayed in the supplementary Fig. 1. ATB-DILI subjects were mainly presented with hepatocellular hepatitis that was characterized by ALT and AST elevation and the onset time of ATB-DILI was different due to individual differences. Based on sample collection time, 9 individuals of each group who were enrolled in the study from November 2019 to December 2020 were assigned to a discovery cohort and age-, gender-, and BMI-matched 18 healthy controls were also involved in the cohort. (Fig. 1A, supplementary Table 8).

**Table 1:** The baseline clinical features of the longitudinal cohort

Clinical features	ATB-DILI group (*n* = 14, 9 + 5)	ATB-Ctrl group (*n* = 14, 9 + 5)	p1	HC group (*n* = 18)	p2	p3
Gender (female, %)	9 (64.29%)	9 (64.29%)	-	10 (55.56%)	-	-
Age (years)	39 ± 11.78	37.64 ± 11.96	0.94	37.72 ± 10.36	0.95	0.99
BMI (kg/m^2)^	23.49 (23.06, 24.12)	22.32 (21.16, 23.6)	0.22	21.87 (21.18, 23.34)	0.04	> 0.999
PTB (n, %)	11 (78.57%)	13 (92.86%)	-	-	-	-
EPTB (n, %)	3 (21.43%)	1 (7.14%)	-	-	-	-
**Baseline clinical indexes**						
ALT (IU/L)	18.5 (14.75, 37.75)	14.5 (11.75, 21.5)	0.30	17 (14, 20.75)	> 0.999	> 0.999
AST (IU/L)	22.21 ± 6.65	20.36 ± 4.36	0.60	18.78 ± 3.92	0.14	0.65
ALP (IU/L)	88.5 ± 38.44	67.64 ± 16.79	0.09	65.78 ± 16.79	0.04	0.98
GGT (IU/L)	27.5 (16.5, 47.5)	15 (10.5, 25.5)	0.10	15.5 (10.75, 27.75)	0.35	> 0.999
TBIL (µmol/L)	10.39 ± 5.16	12.37 ± 4.94	0.56	13.64 ± 4.99	0.18	0.76
WBC (×10^9/L)	6.24 (4.98, 7.76)	6.45 (4.82, 7.61)	> 0.999	5.25 (4.27, 6.32)	0.16	0.18
Hemoglobin (g/L)	141 ± 14.69	134.6 ± 13.08	0.38	143.6 ± 10.61	0.84	0.13
platelet (×10^9/L)	246 ± 78.07	201 ± 35.48	0.08	228 ± 39.67	0.63	0.33
Triglycerides	1.22 (1.06, 2.21)	1.07(0.74, 1.52)	0.48	0.82 (0.70, 1.40)	0.07	> 0.99
Cholesterol	4.69 (4.25, 5.26)	4.47 (3.66, 5.21)	> 0.99	4.55 (4.19, 5.04)	> 0.99	> 0.99
HDL-C	1.11 (0.92, 1.44)	1.33 (1.08, 1.64)	> 0.99	1.56 (1.18, 1.85)	0.04	0.41
LDL-C	2.92 (2.32, 3.57)	2.47 (1.90, 3.50)	0.74	2.66 (2.37, 3.17)	> 0.99	> 0.99

PTB: pulmonary tuberculosis, EPTB: extrapulmonary tuberculosis. The value of *p1* indicates statistical significance between ATB-DILI group and ATB-Ctrl group, *p2* indicates statistical significance between ATB-DILI group and HC group, *p3* indicates statistical significance between ATB-DILI group and ATB-Ctrl group

### ATB-DILI progression is associated with dynamic metabolic changes

A total of 107 samples within the discovery cohort were analyzed for global metabolomics. After quality control and peak identification, we successfully identified 895 metabolites across the different samples. Of these, lipid metabolites account for 46.82% (419/895) which is dominated by fatty acids (FAs) and bile acids (BAs) (48.93%, 205/419) (Fig. 1B). Then, we examined the data globally with PLS-DA, in which the samples were discriminated from the different three groups based on the first two components (Fig. 1C; the PCA results in supplementary Fig. 2).

Next, we performed a comparison of different groups to find statistically significant changed metabolites and data revealed the number of differential metabolites increased along with disease progression in the ATB-DILI group (Supplementary Figs. 3, 4, 5, 6, 7). To identify potential predictive biomarkers of ATB-DILI, we screened the differential metabolites using the following criteria: (1) ATB-DILI T4/T1: fold change (FC) ≥ 1.5 or FC ≤ 0.66, FDR ˂ 0.05, (2) ATB-DILI T4/ATB-Ctrl T4: FC ≥ 1.5 or FC ≤ 0.66, (3) exclusion of exogenous drugs. A total of 77 compounds were obtained, of which 62 metabolites significantly increased at T4 while 15 metabolites decreased. Hierarchical clustering based on the top 77 metabolites revealed that FAs significantly increased at T2, reached their peak at T4, and decreased at T5 while BAs elevated since T3, reached their peak at T4, and remained at a high level at T5 (Fig. 1D).

### Metabolite groups altered during ATB-DILI

**Fig. 2:**
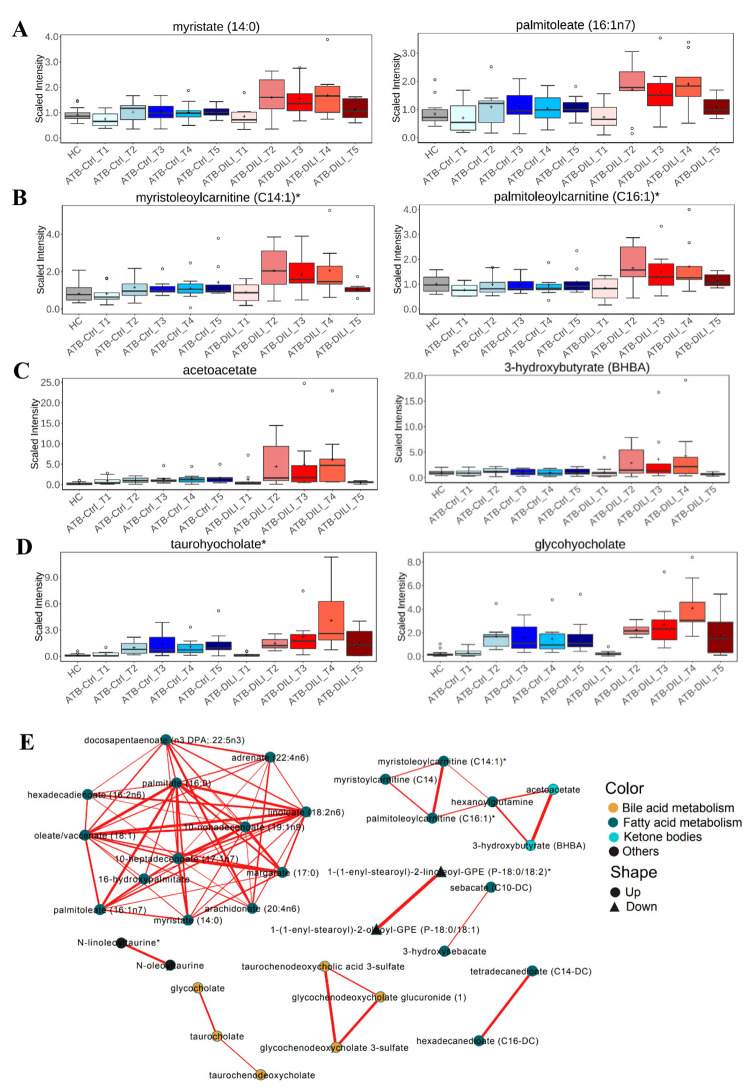
Functional metabolites alter during ATB-DILI. Expression characteristics of long-chain FAs **(A)**, long-chain carnitines **(B)**, ketone body **(C)**, and BAs **(D)** in different groups. The boxes represent the middle 50% of the data, with the line inside the box indicating the median and the cross indicating the mean. The whiskers indicate the maximum and minimum values and circles indicate extreme data point. (E) Weighted correlation network analysis of metabolites. Each node represents a compound, each line represents the correlation between the two compounds, and the line weight represents the correlation coefficients

We further explored the expression characteristics of different subgroups metabolites. The long-chain saturated and unsaturated FAs exhibited a gradual increase at T2-T4 in the ATB-DILI group, with levels higher than those observed in the ATB-Ctrl and HC groups (Fig. 2A). Concurrently, long-chain carnitines that are responsible for FAs transportation from cytoplasm to mitochondrion had also increased at T2-T4 in the ATB-DILI group, and ketone body also exhibited a similar expression pattern (Fig. 2B and C). The phenomenon suggests that FAs transportation, β-oxidation, and ketone body synthesis are enhanced as the disease progresses. Furthermore, we also noted a significant increase in various BAs at T3-T4 in the ATB-DILI group (Fig. 2D), indicating a disturbance in the metabolic balance of BAs as the disease progresses.

Next, we performed correlation analysis on the top 77 metabolites profiles to detect the relationship of functional compounds that changed during the process of ATB-DILI. In supplementary Fig. 8, the top 77 metabolites formed two clusters that mainly belonged to FAs metabolism and BAs metabolism and compounds of the same groups tended to aggregate with each other. We further conducted weighted co-expression network analysis to explore the potential regulatory relationship of metabolites. In Fig. 2E, the largest cluster was composed of a variety of long-chain saturated FAs, monounsaturated FAs, and polyunsaturated FAs. Within the FAs cluster, intra-correlation was remarkably high. The second cluster contained multiple long-chain carnitines and ketone body, which highly correlated with each other. Furthermore, primary and secondary BAs also positively correlated with each other. All these results suggested that metabolic pathways, including long-chain FAs synthesis, elongation, desaturation, β-oxidation, ketone body synthesis, and BAs synthesis, are up-regulated with ATB-DILI occurrence and progression.

### Metabolic dynamics and pathway analysis of ATB-DILI

**Fig. 3:**
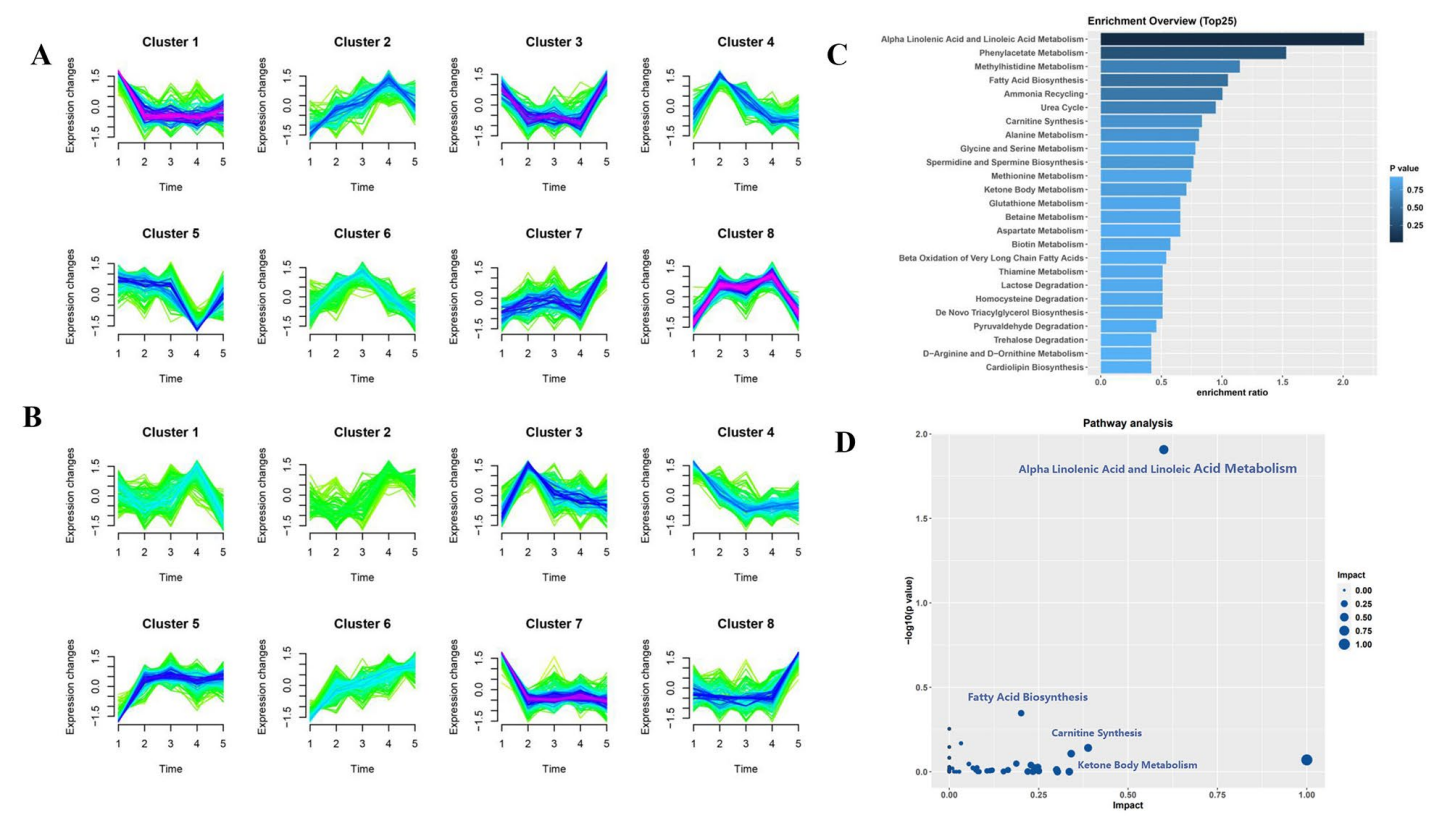
Enrichment, and pathway analysis of the metabolites. **(A-B)** Cluster analysis showed the temporal changes of metabolite intensity during antituberculosis treatment in the ATB-DILI group **(A)** and ATB-Ctrl group **(B)**. **(C-D)** Enrichment and pathway analysis of metabolites that showed significant metabolic inflection point at T4

We longitudinally examined global dynamic changes of all 895 metabolites. There were 8 clusters observed in the ATB-DILI group and ATB-Ctrl group and metabolites of each cluster showed unique metabolic characteristics (Fig. 3A and B). Metabolites that involved in the cluster 2, 3, 5, 7, 8 in the ATB-DILI group showed significant metabolic inflection point at T4, we thus speculated that compounds of the clusters were significantly associated with the occurrence and progression of ATB-DILI. Further enrichment and pathway analysis revealed that alpha linolenic acid and linolenic acid metabolism, fatty acid synthesis, carnitines synthesis, and ketone body metabolism were markedly enriched (Fig. 3C and D). These findings suggested that lipid metabolism and energy metabolic balance might be disrupted by antituberculosis drugs. Under the sustained effect of antituberculosis drugs, BA synthesis, the down-stream metabolic pathway of lipids, could also be significantly up-regulated in the pathological state.

### Prediction biomarkers identification for ATB-DILI

To find candidate biomarkers of ATB-DILI at the early stage, we further applied the random forest (RF) classification algorithm to select the significant variables among the top 77 metabolites. From the longitudinal perspective, we identified metabolites that differentiated samples between multiple timepoints (T2-T4) of disease progression and baseline (T1) in the ATB-DILI group. For this analysis, the prediction accuracy of RF model was 88.89%, 83.33% and 94.44% at T2-T4, respectively. In supplementary Fig. 9A-9 C, top 30 metabolites were listed in order of their importance to the classification scheme. Among them, glycohyocholate (GHCA) and taurohyocholate (THCA) ranked within the top 4 of the lists at different timepoints (T2-T4). Therefore, we speculated that GHCA and THCA could potentially serve as biomarkers for the disease. From the horizontal perspective, we identified compounds that differentiated individuals between ATB-Ctrl group and ATB-DILI group at T2-T4. For this analysis, the prediction accuracy of RF model was 61.11%, 66.67% and 83.33% at T2-T4, respectively. In supplementary Fig. 10A-10 C, we observed that FAs play a major role at T2 and BAs play a vital role at T4. Thus, 5 FAs including myristate (14:0) (MA), palmitoleate (16:1n7) (POA), linoleate (18:2n6) (LA), arachidonate (20:4n6) (ARA), oleate/vaccenate (18:1) (OA) and 6 BAs including glycochenodeoxycholic acid (GCDCA), taurochenodeoxycholic acid (TCDCA), glycocholic acid (GCA), taurocholic acid (TCA), GHCA, and THCA were involved in the following targeted verification (supplementary Table 9).

### Candidate FAs validation in longitudinal cohort and cross-sectional cohort

To validate the predictive capability and universal applicability of candidate biomarkers, we further performed quantitative detection for each biomarker in the longitudinal and cross-sectional cohorts, respectively. Demographics and clinical parameters of the independent cross-sectional cohort are displayed in the Table 2.

**Table 2:** Clinical features of the cross-sectional cohort

Clinical features	CS-DILI group (*n* = 67)	CS-Ctrl group (*n* = 61)	p1	HC group (*n* = 50, 18 + 32)	p2	p3
Gender (female, %)	20 (29.85%)	32 (52.46%)	0.01	30 (60%)	0.001	0.45
Age(years)	38 (28, 51)	28 (23, 40)	0.01	39 (31, 46)	> 0.999	0.003
PTB (n, %)	60 (89.55%)	55(90.16%)	-	-	-	-
EPTB (n, %)	7 (10.45%)	6 (9.84%)	-	-	-	-
**Baseline clinical indexes**						
ALT (IU/L)	166 (125, 254)	10 (8, 15)	˂0.0001	15 (13, 20)	˂0.0001	0.044
AST (IU/L)	128 (87, 207)	17 (15, 22)	˂0.0001	20 (16, 22)	˂0.0001	0.92
ALP (IU/L)	93 (73, 114)	78 (58, 93)	0.001	67 (56, 78)	˂0.0001	0.13
GGT (IU/L)	66 (42, 109.5)	19 (13, 26)	˂0.0001	17 (11, 31)	˂0.0001	> 0.999
TBIL (µmol/L)	9.1 (6.7, 13.6)	8.8 (6.25, 12.8)	> 0.999	11.25 (9.48, 14.5)	0.06	0.01
WBC (×10^9/L)	5.18 (4.45, 7.37)	4.75 (4.22, 5.35)	0.08	5.28 (4.7, 6.5)	> 0.999	0.01
Hemoglobin (g/L)	144 (133, 155)	145 (139, 160)	0.25	142 (133, 156)	> 0.999	0.23
Platelet (×10^9/L)	191 (155, 264)	200 (175, 233)	> 0.999	230 (201, 261)	0.008	0.01
Triglycerides	1.04 (0.77, 1.51)	0.89 (0.73, 1.19)	0.19	0.99 (0.76, 1.44)	> 0.99	0.66
Cholesterol	4.15 (3.49, 4.88)	4.39 (3.98, 5.12)	0.09	4.64 (4.25, 5.24)	0.002	0.322
HDL-C	1.24 (1.00, 1.46)	1.40 (1.18, 1.62)	0.02	1.54 (1.20, 1.79)	< 0.0001	0.20
LDL-C	2.17 (1.74, 2.66)	2.53 (2.03, 3.12)	0.06	2.77 (2.34, 3.37)	< 0.0001	0.04

PTB: pulmonary tuberculosis, EPTB: extrapulmonary tuberculosis. The value of *p1* indicates statistical significance between CS-DILI group and CS-Ctrl group, *p2* indicates statistical significance between CS-DILI group and HC group, *p3* indicates statistical significance between CS-DILI group and ATB-Ctrl group

**Fig. 4:**
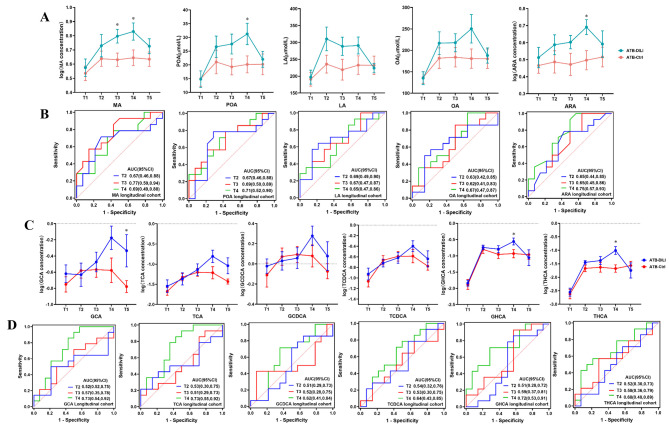
Concentration of candidate biomarkers and ROC analysis in the longitudinal cohort. Plasma concentration **(A)** and ROC analysis **(B)** of 5 FAs in the longitudinal cohort. Plasma concentration **(C)** and ROC analysis **(D)** of 6 BAs in the longitudinal cohort. Data are expressed as mean with SEM **(A, C)**. * indicates *p* < 0.05

In Fig. 4A, we observed that the FAs abundance increased dramatically over time in the ATB-DILI group. To evaluate the ability of 5 FAs to recognize ATB-DILI cases at the early stage, we next performed receiver operating characteristic curve (ROC) analysis at T2-T4, respectively. The area under the curves (AUC) of 5 FAs ranged from 0.63 to 0.69 at T2 and LA had the highest AUC of 0.69; the AUC fluctuated between 0.62 and 0.77 at T3 and MA showed the best performance with AUC of 0.77; the AUC ranged from 0.65 to 0.75 at T4 and ARA had the best diagnostic efficiency with an AUC of 0.75 (Fig. 4B).

**Fig. 5:**
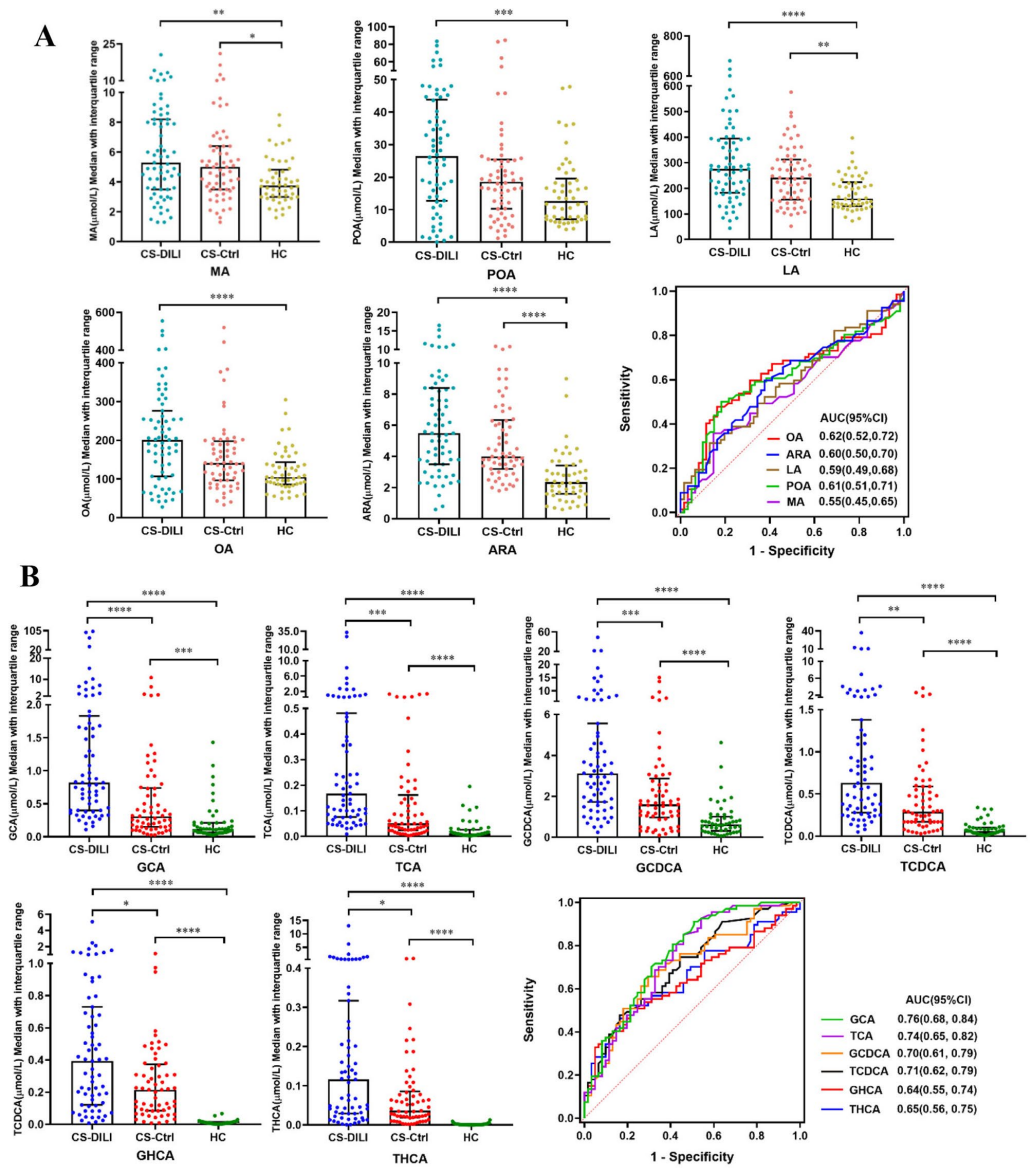
Concentration of candidate biomarkers and ROC analysis in the cross-sectional cohort. Plasma concentration and ROC analysis of 5 FAs **(A)** and 6 BAs **(B)** in the cross-sectional cohort. Data are expressed as median with interquartile range, * indicates *p* < 0.05, ** indicates *p* < 0.01, *** indicates *p* < 0.001, **** indicates *p* < 0.0001

We then explored the abundance changes of FAs in the independent cross-sectional cohort. In Fig. 5A, we observed that the concentrations of FAs in the CS-DILI group were significantly higher than those of the CS-Ctrl group and HC group (*p* < 0.05). The ROC analysis showed that the AUC of OA, POA, ARA, LA, and MA were 0.62, 0.61, 0.60, 0.59, and 0.55, respectively.

These results further validated that the level of FAs is up-regulated during the progression of ATB-DILI. And 5 FAs had the ability to identify ATB-DILI subjects at the early stage (T2, T3) while ALT remained low level (< 3×ULN) (supplementary Fig. 11).

### Candidate BAs validation in longitudinal cohort and cross-sectional cohort

In the ATB-DILI group, the abundance of GCA, TCA, GCDCA, TCDCA was higher than that in ATB-Ctrl group at T4 while the concentrations of GHCA and THCA were higher than those in ATB-Ctrl group at T2-T4 (Fig. 4C). The ROC analysis revealed that 6 BAs had the best diagnostic performance at T4 and the AUCs of GCA, TCA, GCDCA, TCDCA, GHCA, THCA were 0.73, 0.73, 0.62, 0.64, 0.72, and 0.68, respectively (Fig. 4D). The prediction capability of 6 BAs was limited at the early stage (T2, T3) and the AUCs were just in the range of 0.51∼0.59. Consistent with the observation in the longitudinal cohort, the levels of GCA, TCA, GCDCA, TCDCA, GHCA, THCA dramatically increased in CS-DILI group (Fig. 5B). The ROC analysis showed that 6 BAs had a good capability to differentiate patients with ATB-DILI from controls. The AUCs of GCA, TCA, GCDCA, TCDCA, GHCA, THCA were 0.75, 0.74, 0.70, 0.70, 0.64, and 0.65, respectively.

These results further validated that the level of 6 BAs was significantly up-regulated in ABT-DILI subjects. And 6 BAs had a better diagnostic performance when ATB-DILI occurred. We have also tried logistic regression with all or part of biomarker candidates, but the AUC is lower than a single biomarker (data not shown).

## Discussion

ATB-DILI is classified as idiosyncratic hepatotoxicity, which is unpredictable and not dose-dependent. Clinical manifestation varies from transient ALT elevation to acute hepatocellular hepatitis, even acute liver failure. The latency period of the disease ranges from several days to months and all antituberculosis drugs should be stopped whenever the diagnosis is established. However, treatment discontinuation might induce resistance to MTB and further lead to poor outcomes. Therefore, it is necessary to find novel biomarkers that could predict the occurrence of ATB-DILI at the early stage.

In our report, global metabolomics showed the level of multiple long-chain FAs had elevated since T2 and continuously increased at T3-T4 in ATB-DILI subjects. Physiologically, FAs are predominantly metabolized through β-oxidation to provide acetyl-CoA for energy generation via the TCA cycle. During β-oxidation, long-chain FAs are conjugated to carnitine for transport into the mitochondria through the carnitine shuttle. In the mitochondria, the FAs are progressively shortened by two carbons through rounds of β-oxidation, generating NADH, FADH2, and acetyl-CoA (supplementary Fig. 12) [20]. The increase of FAs has been linked to drug-induced hepatic steatosis, possibly due to increased FAs uptake, *de novo* lipogenesis, or reduced utilization [21]. Here, we also observed that the levels of long-chain carnitines significantly increased at T2-T4. These results suggested that increased β-oxidation is occurring in ATB-DILI subjects, possibly as a result of increased levels of free long-chain FAs. In support of this, the ketone body acetoacetate is also significantly elevated in the ATB-DILI subjects. Increased ketogenesis occurs to convert excess acetyl-CoA to water-soluble ketone body and can also be utilized as an indicator of increased β-oxidation (supplementary Fig. 12) [22]. These findings suggest that increased FAs oxidation is occurring in ATB-DILI subjects, possibly as a consequence of increased circulating levels of FAs.

Active lipids could be potential biomarkers to differentiate diseases [21]. In our study, ATB-DILI subjects had a higher level of 5 FAs (MA, POA, OA, LA, ARA) in the longitudinal and cross-sectional cohorts, which indicated abnormal lipid accumulation during the progression of ATB-DILI. Elevated circulating POA indicated that hepatic lipid accumulation was associated with increased *de novo* lipogenesis [23]. High levels of MA, POA, and ARA were significantly correlated with lobular inflammation, ballooning, and fibrosis and could be indicators of hepatic inflammation [24–26].

Untargeted metabolomics also suggested multiple BAs dramatically increased at T2-T4. Physiologically, primary BAs are synthesized in the liver through oxidation of cholesterol and subsequent conjugation with either glycine or taurine before their secretion into the bile. In the intestines, bacteria can deconjugate the primary BAs by removing the glycine and taurine groups, converting them into secondary BAs (supplementary Fig. 13) [27]. BAs serve as signal molecules to regulate glucose, lipid, and energy metabolism by binding to nuclear hormone farnesoid X receptor (FXR) and Takeda G protein receptor 5 (TGR5) [27]. Altered BAs homeostasis would trigger cholestasis, immune disorder, and metabolic disorder, further promoting disease progression [27].

In our report, ATB-DILI subjects had a higher level of 6 BAs (GCA, TCA, GCDCA, TCDCA, GHCA, THCA) both in the longitudinal and cross-sectional cohorts. Recent studies reported that primary BAs (TCA, GCA, TCDCA, GCDCA) were positively correlated with the increased level of bilirubin and greater severity of DILI and high concentration of TCA (≥ 1955.41nmol/L) would predict abnormal liver function after the onset of DILI [28]. Moreover, the animal model of DILI showed that the level of primary BAs (TCA, GCA, TCDCA, GCDCA) differs significantly on day 2 and day 5 after medication and suggested that BAs metabolic disorder is an early event of DILI [29]. Studies have shown that TCA could aggravate cholesterol-induced triglyceride accumulation, thus promoting lipid peroxidation and oxidative stress, finally leading to hepatocyte death [30]. GCDCA and TCA are also able to promote the proliferation and aggregation of activated hepatic stellate cells, further inducing collagen deposition and liver fibrosis [31, 32]. All these results suggested that BAs accumulation might be related to the disease progression.

Additionally, long-time antituberculosis treatment changed gut microorganism diversity and secondary BAs expression. At present, only members of the *Ruminococcaceae* and *Lachnospiraceae* are reported to possess the capability of converting primary BAs to secondary BAs [33]. And decrease of *Ruminococcaceae* has been proven to be closely related to the reduction of deoxycholic acid (DCA) species [33]. However, the synthesis of GHCA and THCA in humans remains to be elucidated. To fully understand the mechanism of BAs changes, further studies need to establish the relationship between gut microbiota and BAs, and then explore the complex interaction between changed microorganisms, BAs, and host.

Overall, our results suggested that the pathway of FAs and BAs metabolism was upregulated as the disease progressed. Among the candidate biomarkers, 5 FAs were able to differentiate ATB-DILI subjects at the early stage (T2 and T3) while 6 BAs had better abilities to identify cases when ATB-DILI occurred (T4). Our report provides a dynamic perspective to understand the pathological process of ATB-DILI. However, our study had its limitations. Firstly, the sample size of untargeted metabolomics is relatively small and candidate biomarker validation was not performed in an independent longitudinal cohort. Thus, the performance of 5 FAs in the prediction of ATB-DILI needed further investigations in a large sample size of longitudinal cohort. Secondly, we included samples of one-third course and two-thirds course for biomarker screen but this practice might limit its clinical application in the future. Thirdly, we have tried the logistic regression algorithm with all or part biomarker candidates, but the AUC is lower than a single biomarker. Relying on a single biomarker to identify ATB-DILI subjects is challenging in real clinical settings. Finally, although we have established associations between lipid metabolism and ATB-DILI, we have not explored its biological mechanism. Further studies are warranted to explore the underlying mechanisms.

### Electronic supplementary material

Below is the link to the electronic supplementary material.

Supplementary Material 1

Supplementary Material 2

Supplementary Material 3

Supplementary Material 4

## Data Availability

We provide the normalized and imputed data as supplementary materials for subsequent research. Any other data supporting our findings will be made available from the corresponding author on reasonable request.
