# Supplementary material for: Longitudinal metabolomics of human plasma reveal metabolic dynamics and predictive markers of antituberculosis drug-induced liver injury
Source: Respir Res. 2024 Jun 21;25:254. doi: 10.1186/s12931-024-02837-8 (PMC11193241; doi:10.1186/s12931-024-02837-8)
Supplement: Supplementary file 1 — Supplementary Material 1 [file 12931_2024_2837_MOESM1_ESM.pdf]

Supplementary materials

Untargeted metabolomics analysis

All procedures were performed at Discovery HD4^{TM} Platform[1]. Plasma (100 μl) was treated with 450 μl methanol containing internal standards for quality control purposes. Then, the mixtures were vigorously shaken for 2 min followed by centrifugation, thus proteins were precipitated and small molecules were released. The resulting supernatants were divided into five fractions: two for analysis by two separate reverse phase (RP) ultraperformance liquid chromatography/tandem mass spectrometry (UPLC-MS/MS) methods with positive ion mode electrospray ionization (ESI), one for analysis by RP/UPLC-MS/MS with negative ion mode ESI, one for analysis by hydrophilic-interaction chromatography (HILIC) UPLC-MS/MS with negative ion mode ESI, and one was reserved for backup. Samples were placed briefly on a TurboVap® (Zymark) to remove the organic solvent. The sample extracts were stored overnight under nitrogen before preparation for analysis.

All methods utilized a Waters ACQUITY ultra-performance liquid chromatography and a Thermo Scientific Q-Exactive high resolution/accurate mass spectrometer interfaced with a heated electrospray ionization (HESI-II) source and Orbitrap mass analyzer operated at 35,000 mass resolution. The sample extract was dried then reconstituted in solvents compatible to each of the four methods. Each reconstitution solvent contained a series of standards at fixed concentrations to ensure injection and chromatographic consistency. One aliquot was analyzed under positive electron spray ionization (ESI), chromatographically optimized for more hydrophilic compounds. In this method, the extract was gradient eluted from a C18 column (Waters UPLC BEH C18-2.1x100 mm, 1.7 μm) using water and methanol, containing 0.05% perfluoropentanoic acid (PFPA) and 0.1% formic acid. Another aliquot was also analyzed under ESI positive mode, and it was chromatographically optimized for more hydrophobic compounds. In this method, the extract was gradient eluted from the same afore mentioned C18 column using methanol, acetonitrile, water, 0.05% PFPA and 0.01% formic acid and was operated at an overall higher organic content. The third aliquot was analyzed using negative ESI mode optimized conditions using a separate dedicated C18 column. The

basic extracts were gradient eluted from the column using methanol and water, however with 6.5 mM Ammonium Bicarbonate at pH 8. The fourth aliquot was analyzed using negative ESI mode following elution from a HILIC column (Waters UPLC BEH Amide 2.1x150 mm, 1.7 µm) using a gradient consisting of water and acetonitrile with 10 mM Ammonium Formate, pH 10.8. The MS analysis alternated between MS and data-dependent MSn scans using dynamic exclusion. The scan range varied slightly between methods but covered 70-1000 m/z.

Several types of controls were analyzed in concert with the experimental samples: a pooled matrix sample generated by taking a small volume of each experimental sample served as a technical replicate throughout the data set; extracted water samples served as process blanks; and a cocktail of quality control (QC) standards that were carefully chosen not to interfere with the measurement of endogenous compounds were spiked into every analyzed sample, allowed instrument performance monitoring and aided chromatographic alignment. Instrument variability was determined by calculating the median relative standard deviation (RSD) for the standards that were added to each sample prior to injection into the mass spectrometers. The overall process variability was determined by calculating the median RSD for all endogenous metabolites present in 100% of the pooled matrix samples. Experimental samples were randomized across the platform run with QC samples spaced evenly among the injections.

Raw data was extracted, peak-identified and QC processed using Discovery HD4TM's hardware and software. Compounds were identified by comparison to library entries of purified standards or recurrent unknown entities. Discovery HD4^{TM} maintains a library based on authenticated standards that contains the retention time/index (RI), mass to charge ratio (m/z), and chromatographic data (including MS/MS spectral data) on all molecules present in the library. Biochemical identifications are based on three criteria: retention index within 200ms of the proposed identification, accurate mass match to the library within (-5ppm, 5ppm), and the MS/MS forward and reverse scores between the experimental data and authentic standards. The MS/MS scores are based on a comparison of the ions present in the experimental spectrum to the ions present in the library spectrum. All identification results generated by software were manually

double-checked for the accuracy. The dataset was normalized with median and missing values were imputed with minimum values.

Targeted metabolomics analysis

Quantitative analysis of fatty acids

FAs were quantitatively measured according to the commercial kit instruction (Qlife, China). An aliquot of 50 μL plasma sample was mixed with 450 μL working solution containing internal standards. The mixture was vigorously shaken for 5 min, then centrifuged at 12000 rpm at 4°C for 5 min. The supernatant was transferred to vials for UPLC-MS/MS analysis. Simultaneously, a series of standard calibration samples and QC samples were pretreated for calibration curve and quality assurance.

The UPLC-MS/MS system consisted of a Waters AUQUITY UPLC and a Waters XEVO TQS instrument equipped with an ESI source (Waters, the United States of America). All chromatographic separation solution was prepared according to kit instruction. Phase A was diluted 100 times with water and phase B was diluted 100 times with the mixture of acetonitrile and isopropanol at 1:1. The extracts were gradient-eluted from C18 column (Waters UPLC BEH C18-2.1x100 mm, 1.7 μm). The flow rate was 0.3 mL/min with the following mobile phase gradient: 0-2.7min (73%B), 2.7-4.5min (98%B), 4.5-6.5min (73%B). The column was maintained at 40°C and the injection volume for all samples was 1μL. The extracts were analyzed under negative ESI mode and the data were collected with multiple reaction monitor (MRM).

Quantitative analysis of bile acids

BAs were quantified using commercial kit-based method and in-house established method.

Commercial kit-based method: A total of 4 BAs were quantitatively measured according to the commercial kit instruction (Qlife, China). An aliquot of 50 μL plasma sample was mixed with 200μL working solution containing internal standards. The mixture was vigorously shaken for 5 min, then centrifuged at 12000rpm at 4°C for 5 min. The supernatant was transferred to a 96-well plate for UPLC-MS/MS analysis. Simultaneously, a series of standard calibration samples and QC samples were pretreated for calibration curve and quality assurance.

The UPLC-MS/MS system consisted of a Waters AUQUITY UPLC and a Waters XEVO TQS instrument equipped with an ESI source (Waters, the United States of America). All chromatographic separation solution was prepared according to kit instruction. Phase A was diluted 100 times with water and phase B was diluted 100 times with acetonitrile. The extracts were gradient-eluted from C18 column (Waters UPLC BEH C18-2.1x50 mm, 1.7 μm). The flow rate was 0.4 mL/min with the following mobile phase gradient: 0-3.5 min (35%B), 3.5-3.8 min (98%B), 4.5-5 min (35%B). The column was maintained at 40°C and the injection volume for all samples was 2μL. The extracts were analyzed under negative ESI mode and the data were collected with MRM.

in-house established method: Taurohyocholate (THCA) and glycohyocholic acid (GHCA) were quantitatively measured using in-house established method. The method in the study was developed and optimized on the basis of previously reported method[2]. A series of standard calibration samples and QC samples were diluted with desalted plasma (depleted of bile acids using activated charcoal) for calibration curve and mixed standards were prepared using desalted plasma for quality assurance. An aliquot of 100 μL was mixed with 600 μL acetonitrile–methanol (8:2 v/v) containing internal standards. The mixture was shaken at 20°C at 1500rpm for 20min, and was then centrifuged at 13000×g at 4°C for 30min. An aliquot of 600μL supernatant was transfer to another tube for vacuum drying. The sample extracts were reconstituted in 25 μL acetonitrile–methanol (9/1, v/v) containing 0.01% formic acid and vortexed at 10°C at 1500 rpm for 20min. Then 25 μL water containing 0.01% formic acid was added and re-vortexed at 10°C at 1500 rpm for 20 min. After that, the sample was centrifuged at 4°C at 1500 rpm for 20min and the supernatant was transferred to a vial for UPLC-MS/MS.

The UPLC-MS/MS system consisted of a LC30 (SHIMADZU, Japan) and a QTRAP®6500 instrument (SCIEX, the United States of America). Mobile phase consisted of water with 0.01% formic acid (phase A) and acetonitrile/methanol with 0.01% formic acid (9/1, v/v, phase B). The extracts were gradient-eluted from C18 column (Waters UPLC BEH C18-2.1x100 mm, 1.7 μm). The flow rate was 0.4 mL/min

with the following mobile phase gradient: 0-6min (30-99%B), 6-7 min (99%B), 7-11min (30%B). The column was maintained at 40℃ and the injection volume for all samples was 3 μL. The extracts were analyzed under negative ESI mode and the data were collected with MRM.

The extraction recovery, accuracy, precision, limit of detection (LOD), and limit of quantification (LOQ) of the method were evaluated and the results were shown in supplementary Table3,4,5,6.

Supplementary reference

[1] Shen B., Yi X., Sun Y., et al. Proteomic and metabolomic characterization of covid-19 patient sera[J]. Cell, 2020, 182(1): 59-72 e15.

[2] Zheng X., Chen T., Zhao A., et al. Hyocholic acid species as novel biomarkers for metabolic disorders[J]. Nat Commun, 2021, 12(1): 1487.
