# Supplementary material for: Longitudinal metabolomics of human plasma reveal metabolic dynamics and predictive markers of antituberculosis drug-induced liver injury
Source: Respir Res. 2024 Jun 21;25:254. doi: 10.1186/s12931-024-02837-8 (PMC11193241; doi:10.1186/s12931-024-02837-8)
Supplement: Supplementary file 2 — Supplementary Material 2 [file 12931_2024_2837_MOESM2_ESM.pdf]

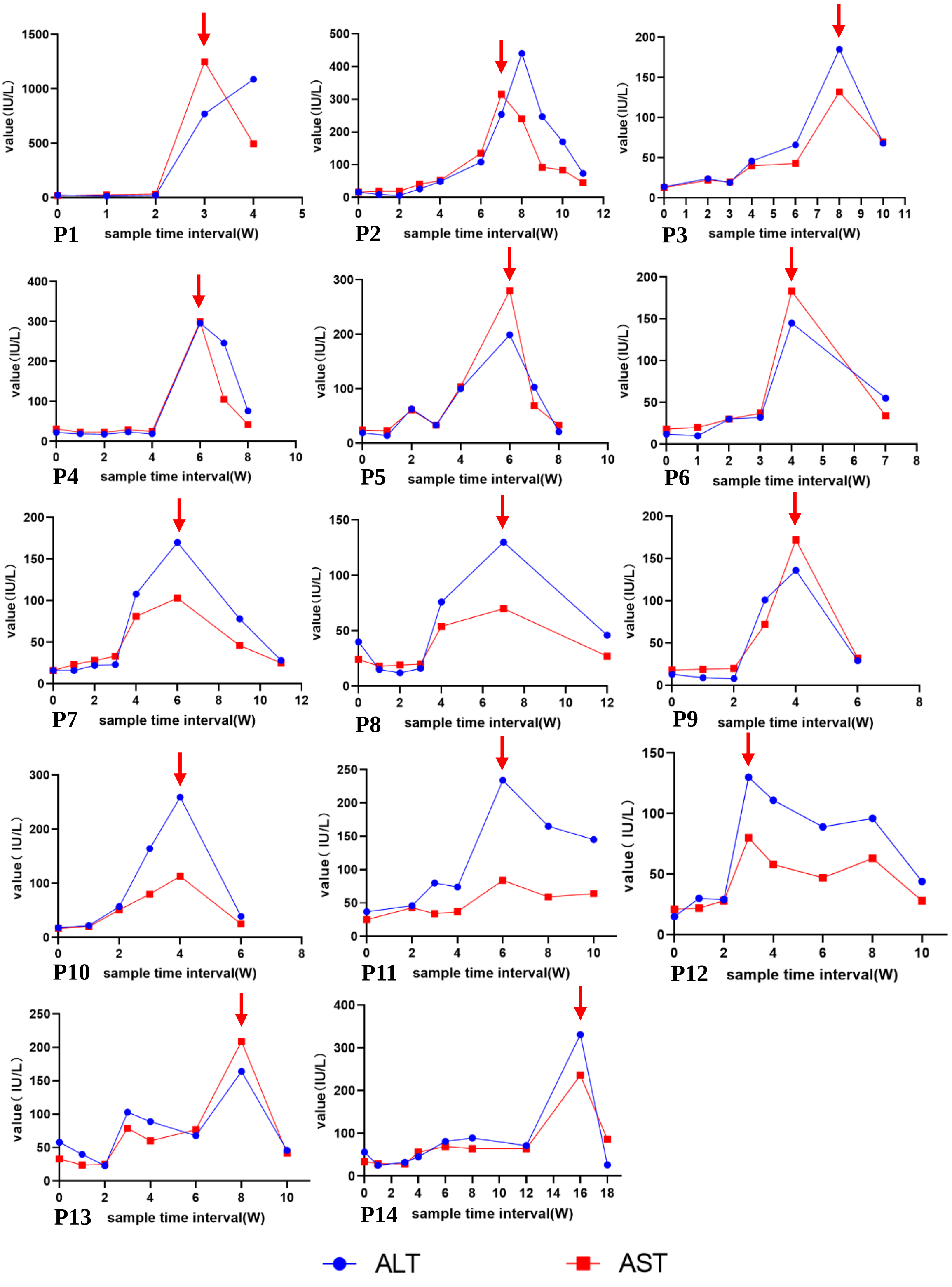

Supplementary figure 1: Dynamic changes of ALT and AST from baseline to the occurrence of ATB-DILI. Among the patients, P1-P9 were assigned to the discovery cohort for untargeted metabolomics detection. Additionally, P14 received a standard regime (HL2ZE) treatment for the first two months and a new therapeutic regime with prothionamide, rifapentine, pyrazinamide, and moxifloxacin for the next two months

because the phenotype drug susceptibility test indicated isoniazid and ethambutol were resistant. The patient stopped pyrazinamide since week sixteen of antituberculosis treatment, then ALT and AST decreased and recovered to baseline level. Thus, the patient was included in the ATB-DILI group for targeted validation.

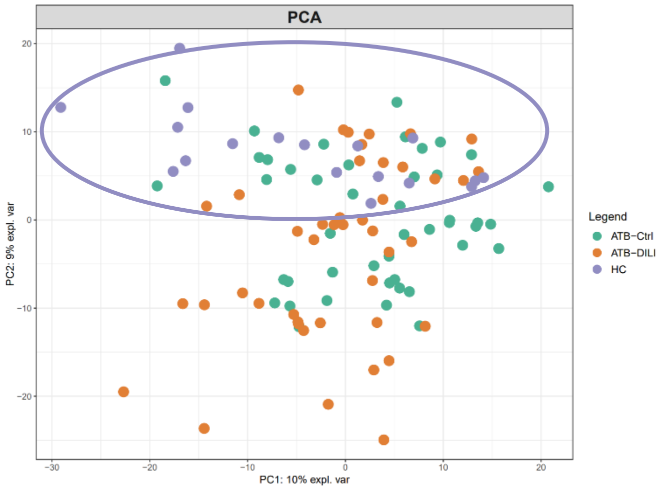

Supplementary figure 2: the principal component analysis: only HC group was differentiated from the three groups.

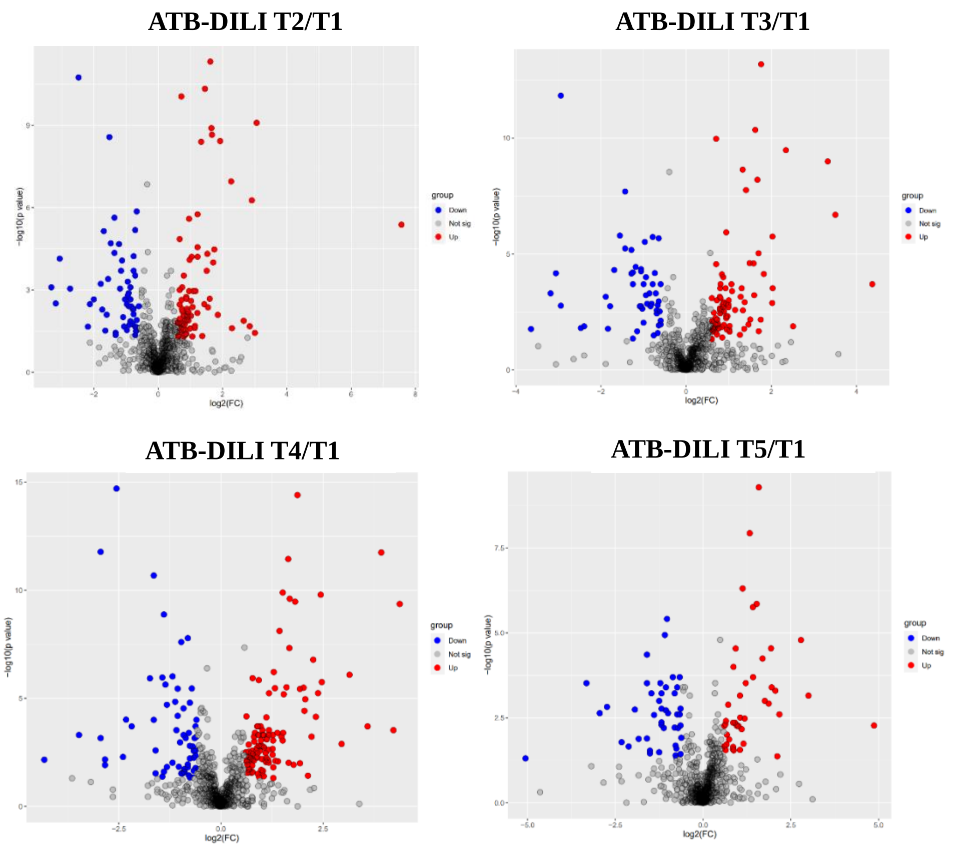

Supplementary figure3: volcano plot of the different timepoints in the ATB-DILI group

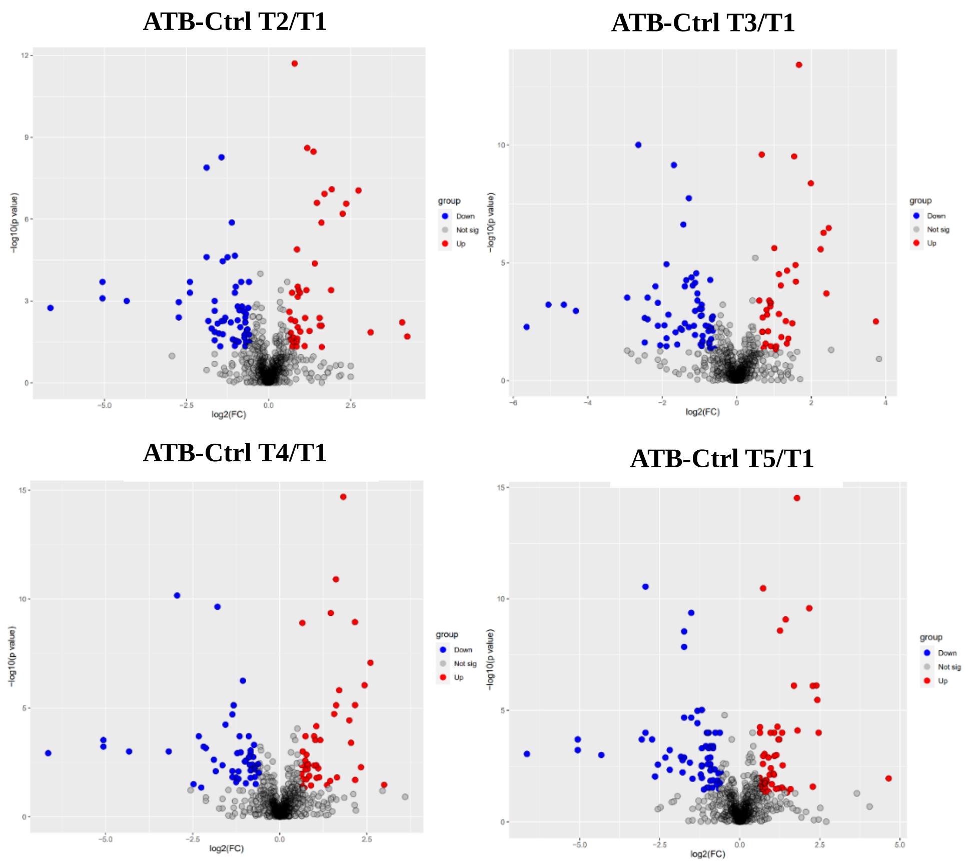

Supplementary figure4: volcano plot of the different timepoints in the ATB-Ctrl group

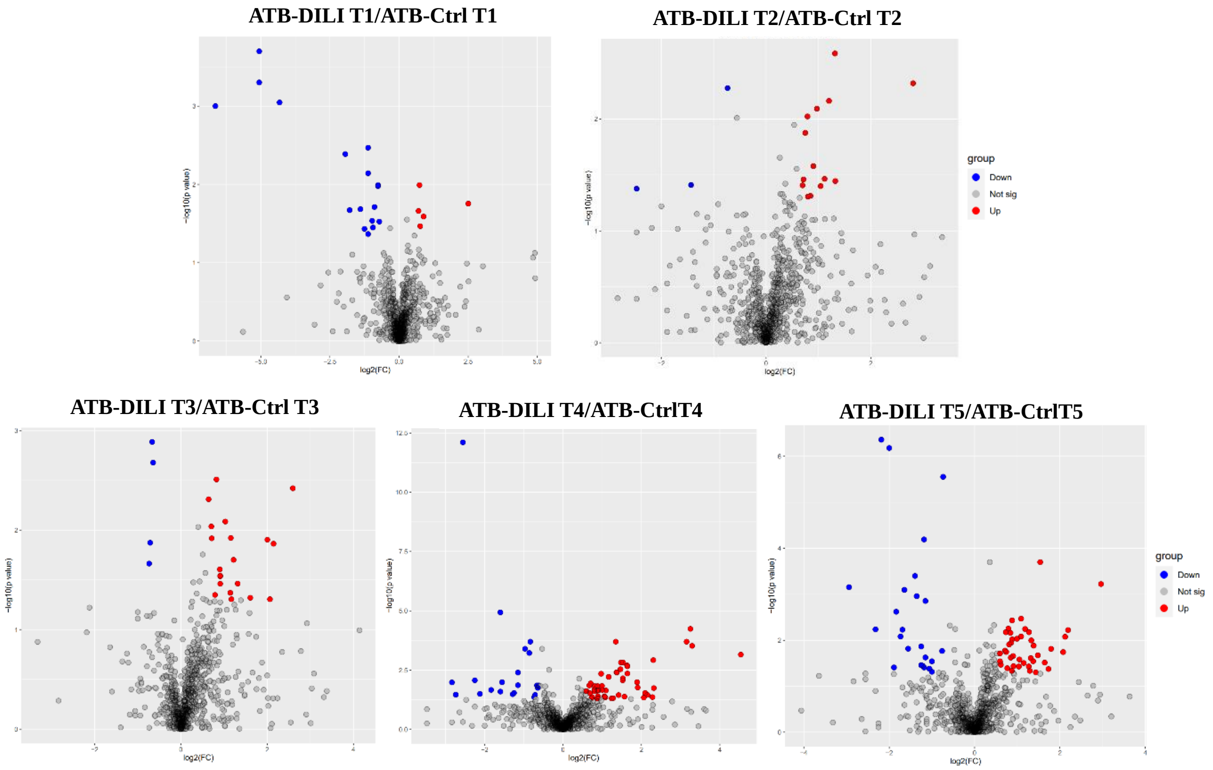

Supplementary figure5: side-by-side volcano plot of multiple timepoints in the ATB-DILI and ATB-Ctrl group

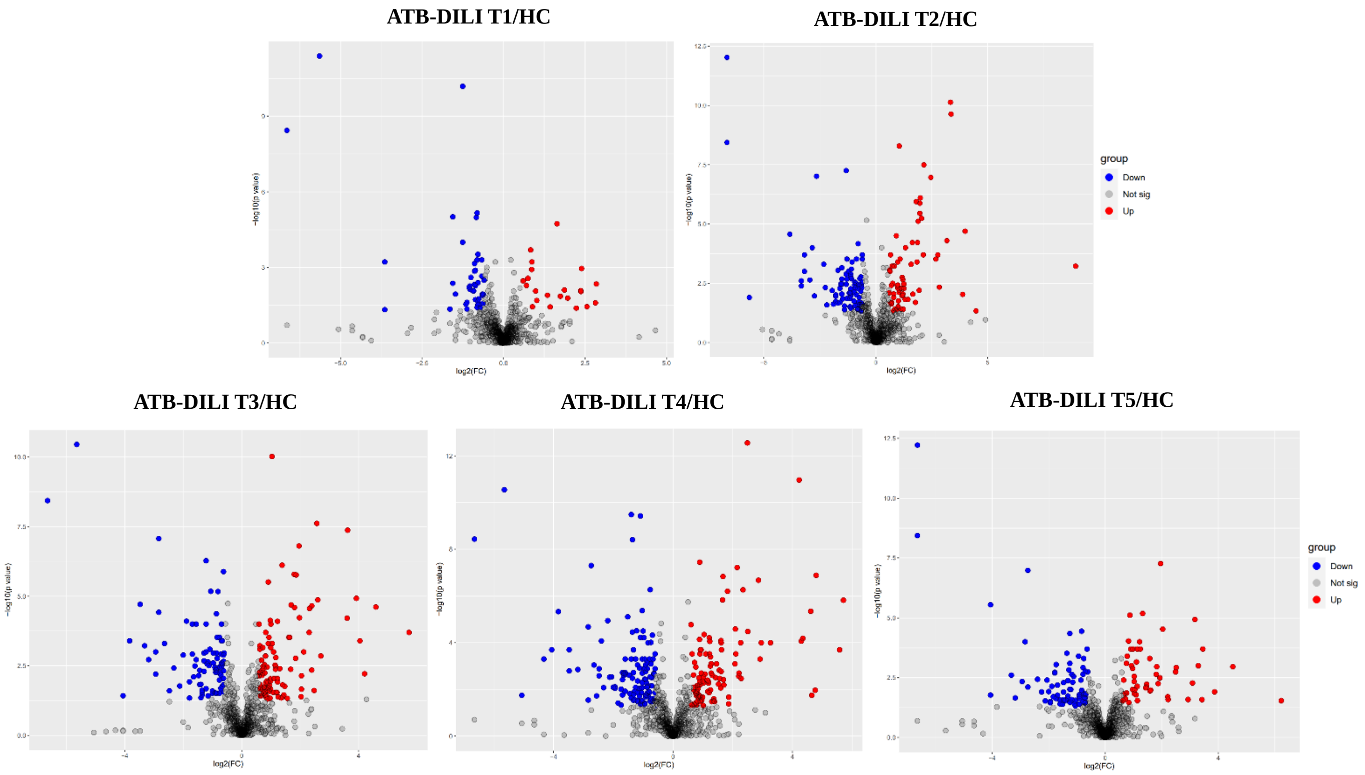

Supplementary figure6: volcano plot of the different timepoints in the ATB-DILI group with HC group

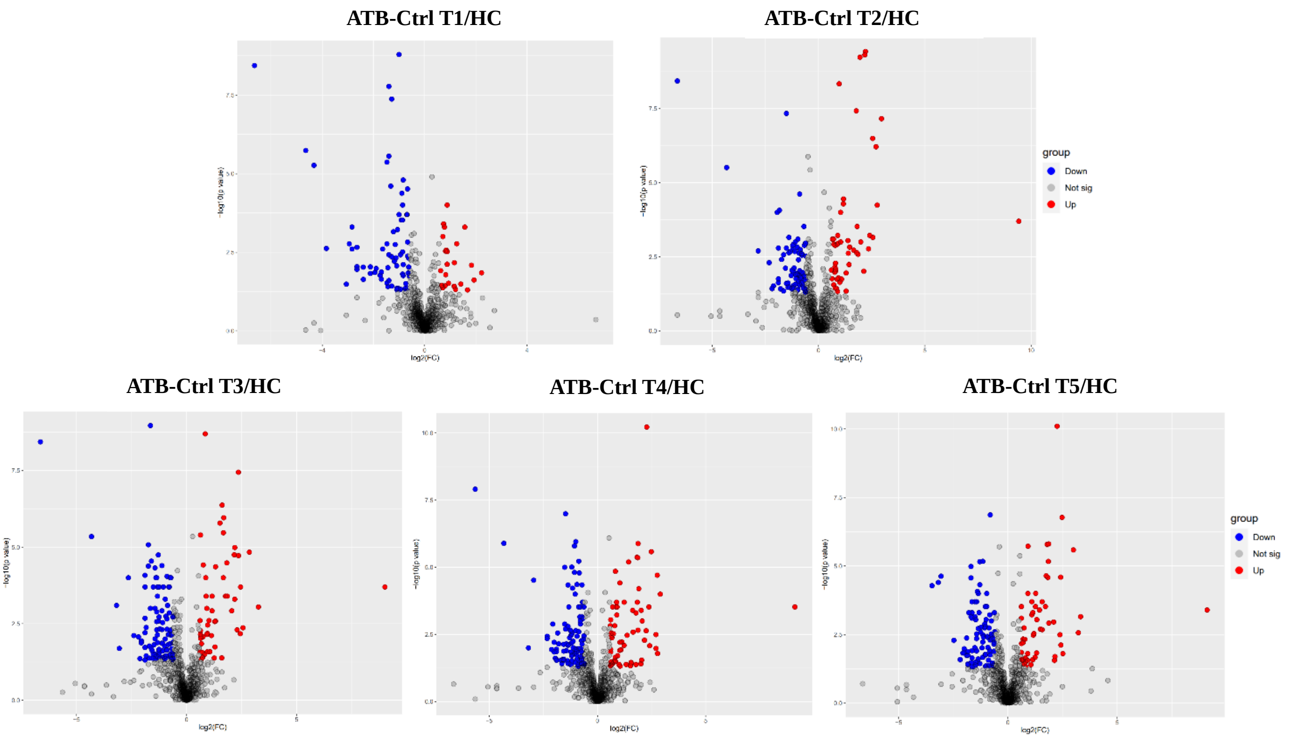

Supplementary figure7: volcano plot of the different timepoints in the ATB-Ctrl group with HC group

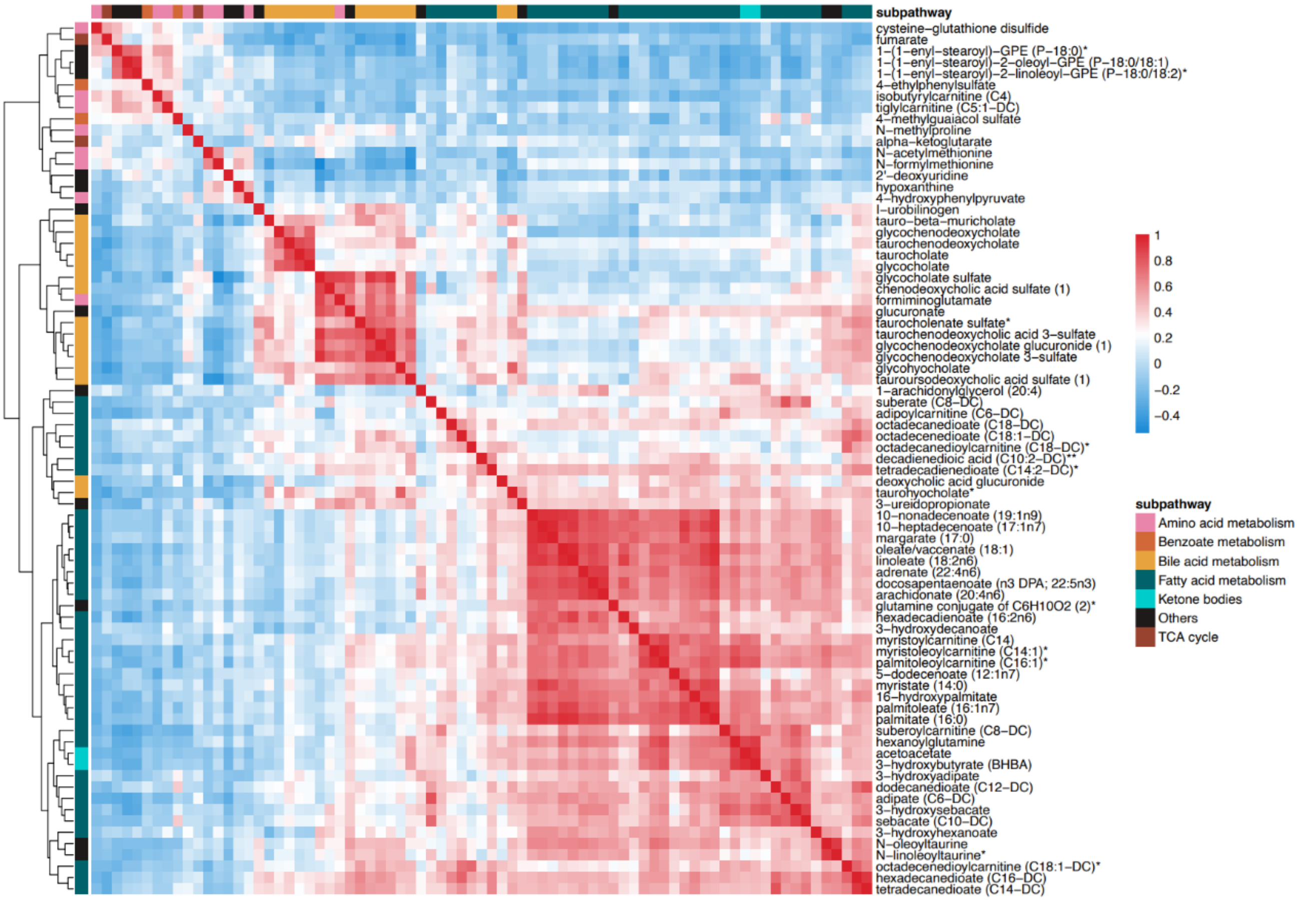

Supplementary figure8: correlation matrix colored by Pearson correlation coefficient

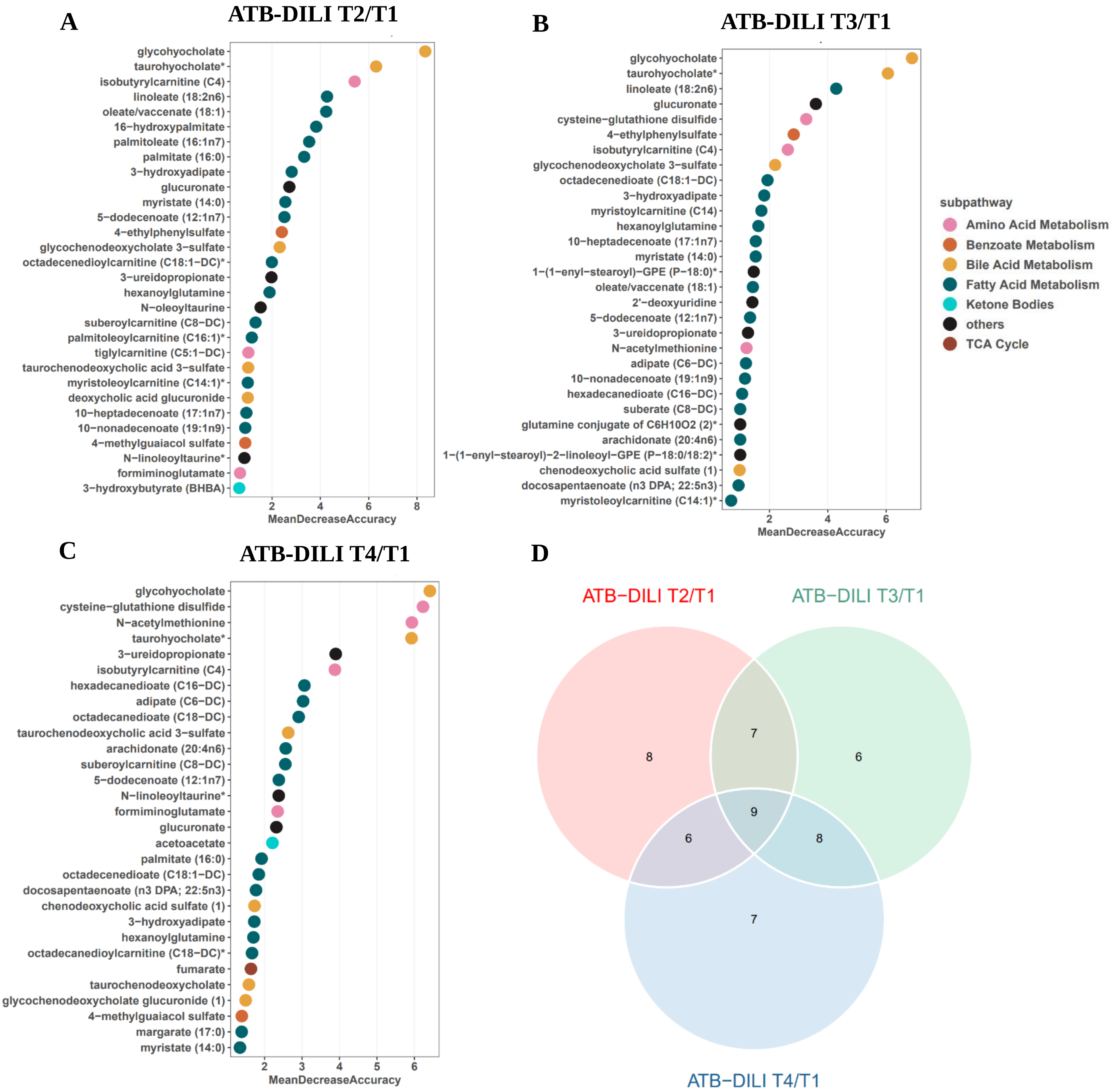

Supplementary Figure9: Random Forest analysis of ATB-DILI T2-T4 versus T1 (A-C) Top30 compounds that contributed to RF classification analysis at T2-T4 in the ATB-DILI group. (D) Venn diagram showing shared and unique metabolites that contributed to RF analysis.

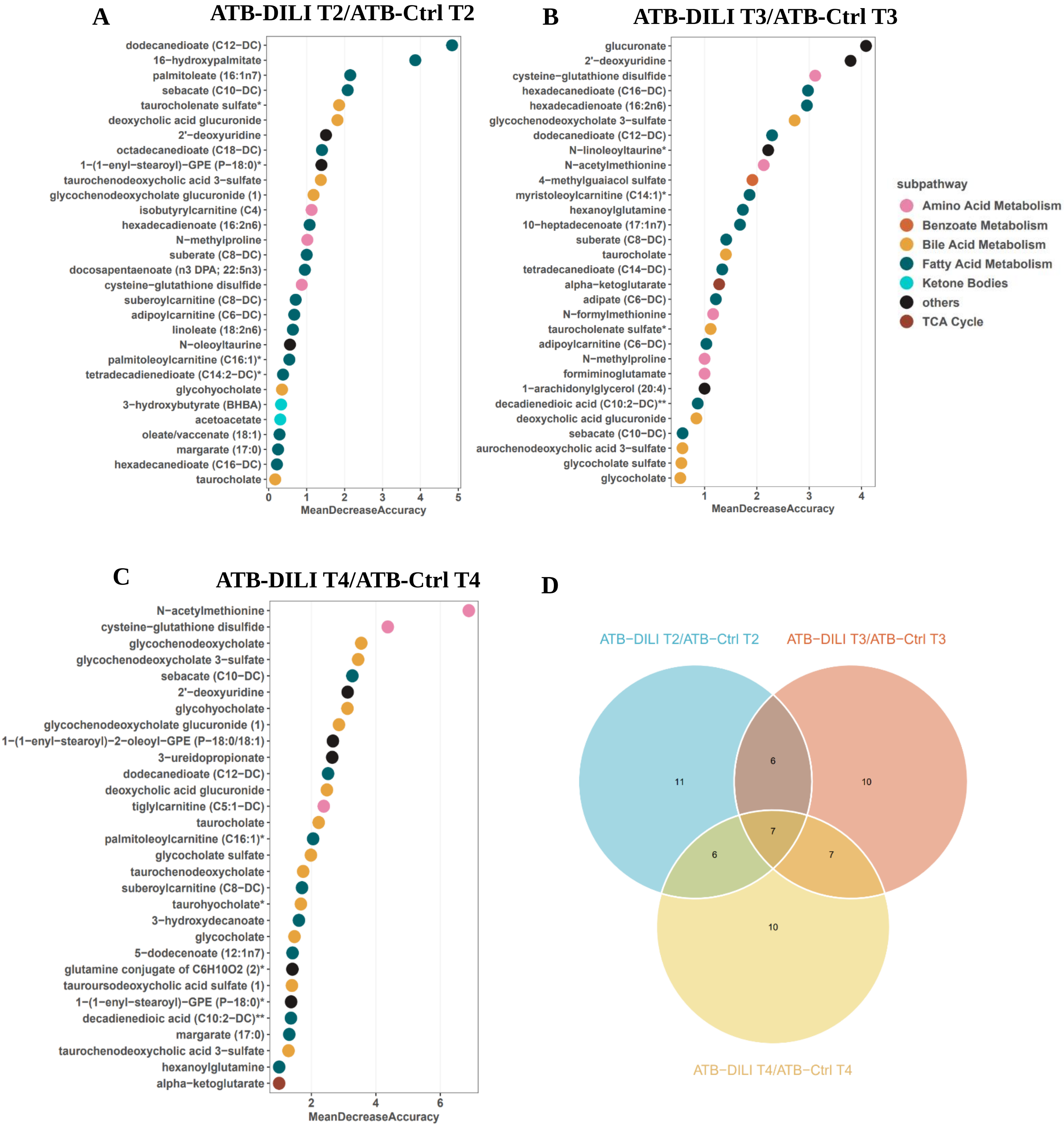

Supplementary Figure10: Random Forest analysis of ATB-DILI T2-T4 versus ATB-Ctrl T2-T4

(A-C) Top30 compounds that contributed to RF classification between the ATB-DILI and ATB-Ctrl group at T2-T4. (D) Venn diagram showing shared and unique metabolites that contributed to RF analysis.

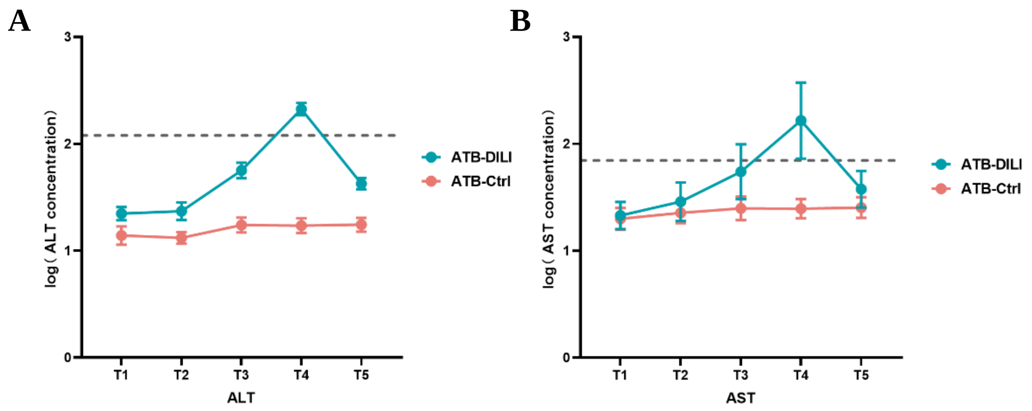

Supplementary Figure11: Comparisons of ALT and AST between the ATB-DILI group and ATB-ctrl group

(A) The black dotted line presented 3×ULN of ALT.

(B) The black dotted line presented 2×ULN of AST.

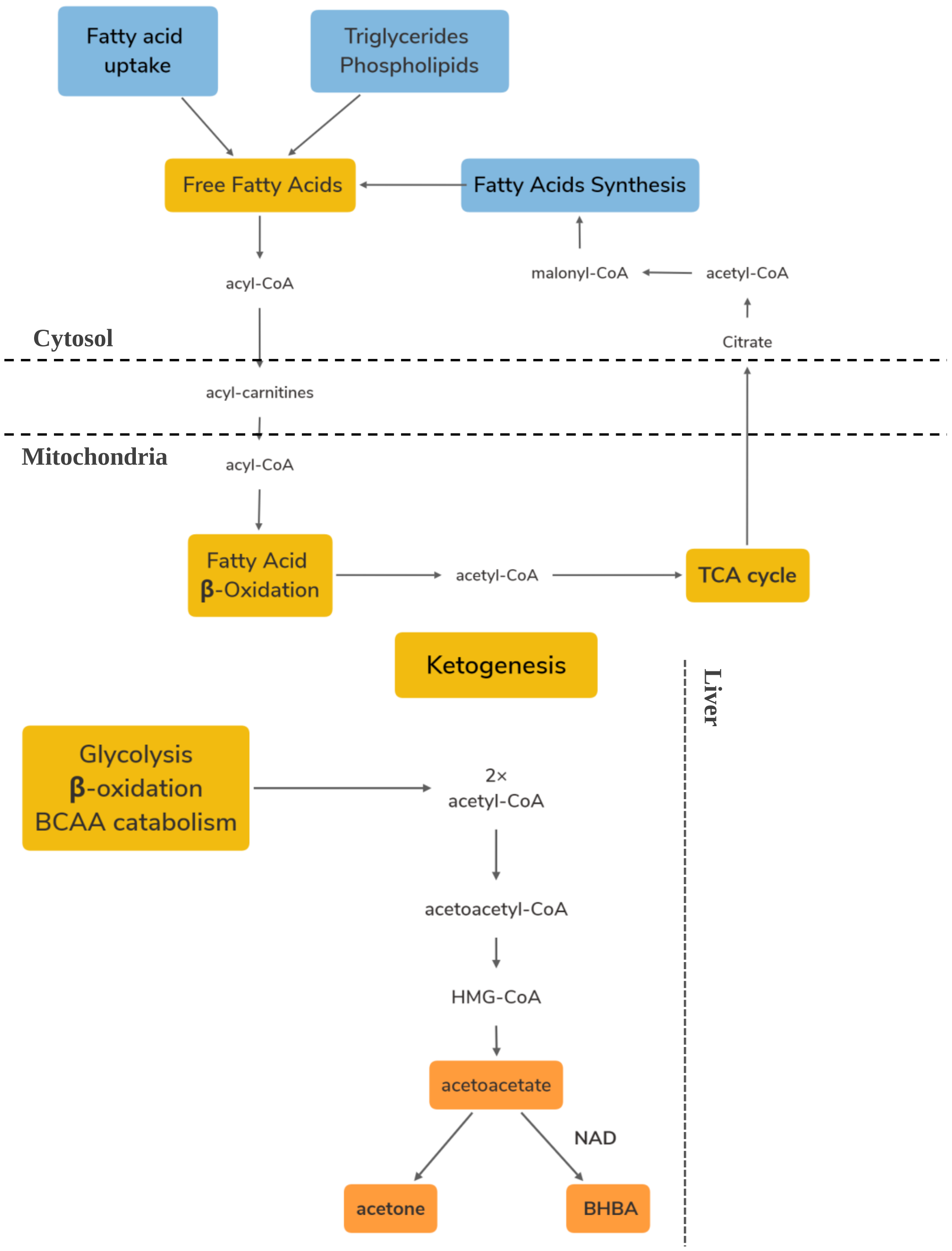

Supplementary figure12: Pathway of fatty acids and ketone body metabolism

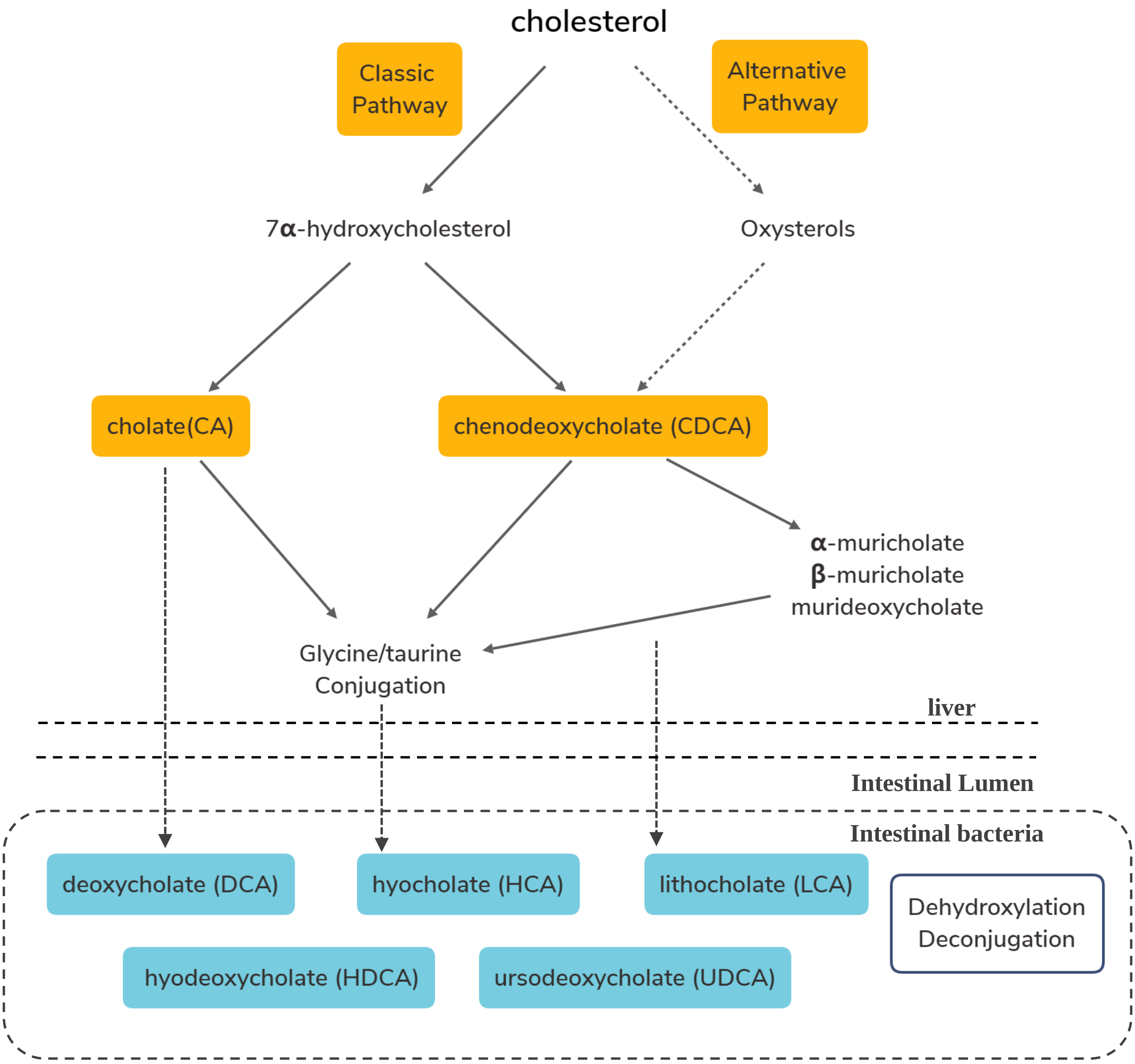

Supplementary figure13: Pathway of bile acids metabolism
