# Supplementary material for: Longitudinal metabolomics of human plasma reveal metabolic dynamics and predictive markers of antituberculosis drug-induced liver injury
Source: Respir Res. 2024 Jun 21;25:254. doi: 10.1186/s12931-024-02837-8 (PMC11193241; doi:10.1186/s12931-024-02837-8)
Supplement: Supplementary file 4 — Supplementary Material 4 [file 12931_2024_2837_MOESM4_ESM.docx]

**Supplementary Table1 fatty acids and matched internal standards**

| **Fatty acids** | **Ion pair** | **internal standards** | **Ion pair** |
| --- | --- | --- | --- |
| MA | 227.2>227.21 | DHA-IS | 332.296>288.323 |
| POA | 253.2>253.21 | DHA-IS | 332.296>288.323 |
| LA | 279.2>279.21 | DGLA-IS | 311.31>311.31 |
| ARA | 303.336>205.254 | DHA-IS | 332.296>288.323 |
| OA | 281.2>281.2 | D3-PA | 258.2>258.2 |

MA: myristic acid, POA: palmitoleic acid, LA: linoleic acid, ARA: arachidonic acid, OA: oleic acid. DHA: docosahexaenoic acid, DGLA: dihomo-γ-linolenic acid, PA: palmitic acid.

**Supplementary Table2 bile acids and matched internal standards**

| **Bile acids** | **Ion pair** | **Internal standards** | **Ion pair** |
| --- | --- | --- | --- |
| TCDCA | 498.3>80.1 | D5-TCDCA | 503.3>79.9 |
| GCDCA | 448.3>73.95 | D5-GDCA | 453.3>73.95 |
| GCA | 464.3>73.95 | D5-GCA | 469.3>73.95 |
| TCA | 514.35>80.01 | D5-TCA | 519.3>79.9 |
| GHCA | 464.3>74.1 | D4-GLCA | 436.3>74.1 |
| THCA | 514.0>80.0 | D4-TCA | 518.3>80.0 |

GCDCA: glycochenodeoxycholic acid, TCDCA: taurochenodeoxycholic acid, GCA: glycocholic acid, TCA: taurocholic acid, GHCA: glycohyocholic acid, THCA: taurohyocholate.

**Supplementary Table3 The extraction recovery of GHCA, THCA, and HCA**

| **Bile acids** | **Extraction recovery（%）** | | | |
| --- | --- | --- | --- | --- |
|  | **Low concentration** | **Median concentration** | **High concentration-1** | **High concentration-2** |
| GHCA | 70.34 | 84.55 | 84.01 | 80.67 |
|  | 76.58 | 86.10 | 83.47 | 85.54 |
|  | 70.98 | 80.40 | 85.62 | 86.63 |
|  | 71.09 | 80.88 | 82.08 | 87.99 |
| THCA | 74.47 | 88.02 | 85.56 | 80.81 |
|  | 80.64 | 89.65 | 84.15 | 87.74 |
|  | 74.13 | 83.87 | 84.46 | 87.77 |
|  | 76.83 | 87.12 | 85.20 | 89.11 |
| HCA | 70.24 | 81.87 | 80.44 | 80.52 |
|  | 77.08 | 83.14 | 80.41 | 80.85 |
|  | 73.58 | 80.15 | 80.26 | 82.01 |
|  | 74.92 | 81.05 | 80.79 | 84.94 |

Each concentration was repeated 4 times in parallel.

**Supplementary Table4 The precision and accuracy of GHCA**

| **Concentration**（nmol/L） | **High** | **Median** | **Low** | **LOQ** | **LOD** |
| --- | --- | --- | --- | --- | --- |
| Measuring concentration | 359.7 | 52.0 | 5.27 | 0.48 | - |
|  | 346.3 | 48.3 | 5.51 | 0.43 | - |
|  | 359.6 | 46.8 | 5.63 | 0.44 | - |
|  | 348.6 | 47.4 | 5.55 | 0.51 | - |
|  | 360.7 | 49.4 | 5.36 | 0.42 | - |
|  | 349.3 | 46.1 | 5.62 | 0.44 | - |
| Theoretical concentration | 400 | 50 | 6.25 | 0.5 | 0.2 |
| Average concentration | 354.0 | 45.5 | 5.52 | 0.46 | - |
| Accuracy（%） | 88.5 | 96.7 | 87.8 | 90.7 | - |
| RSD（%） | 1.87 | 4.39 | 2.34 | 7.36 | 3.71 |

**Supplementary Table5 The precision and accuracy of THCA**

| **Concentration**（nmol/L） | **High** | **Median** | **Low** | **LOQ** | **LOD** |
| --- | --- | --- | --- | --- | --- |
| Measuring concentration | 343.2 | 49.2 | 5.4 | 0.45 | - |
|  | 342.7 | 45.5 | 5.4 | 0.44 | - |
|  | 351.5 | 44.5 | 5.5 | 0.43 | - |
|  | 336.4 | 44.7 | 5.6 | 0.44 | - |
|  | 339.2 | 44.9 | 5.4 | 0.47 | - |
|  | 346.8 | 44.1 | 5.6 | 0.44 | - |
| Theoretical concentration | 400 | 50 | 6.25 | 0.5 | 0.2 |
| Average concentration | 343.3 | 45.5 | 5.47 | 0.46 | - |
| Accuracy（%） | 85.8 | 91.0 | 87.7 | 89.0 | - |
| RSD（%） | 1.56 | 4.11 | 2.01 | 3.26 | 5.78 |

**Supplementary Table6 The precision and accuracy of HCA**

| **Concentration**（nmol/L） | **High** | **Median** | **Low** | **LOQ** | **LOD** |
| --- | --- | --- | --- | --- | --- |
| Measuring concentration | 327.1 | 42.9 | 5.75 | 0.42 | - |
|  | 321.9 | 41.5 | 5.53 | 0.45 | - |
|  | 343.1 | 40.3 | 5.66 | 0.44 | - |
|  | 353.4 | 40.9 | 5.43 | 0.43 | - |
|  | 324.8 | 43.7 | 5.48 | 0.47 | - |
|  | 330.3 | 42.2 | 5.54 | 0.45 | - |
| Theoretical concentration | 400 | 50 | 6.25 | 0.5 | 0.2 |
| Average concentration | 333.4 | 41.92 | 5.56 | 0.45 | - |
| Accuracy（%） | 83.4 | 84 | 89 | 88.7 | - |
| RSD（%） | 1.78 | 3.06 | 2.14 | 3.83 | 6.19 |

**Supplementary Table7 Therapeutic regimes of each patient in the ATB-DILI group**

| **Patients** | **Age(years)** | **gender** | **diagnosis** | **Initial therapeutic regimes** | **Clinical interventions after**  **ATB-DILI** | **Restart therapeutic regimes** |
| --- | --- | --- | --- | --- | --- | --- |
| P1 | 57 | male | PTB | INH, RIF, EMB, PZA | Stopped all anti-TB drugs | none |
| P2 | 28 | female | PTB | INH, RFT, EMB, PZA | Stopped all anti-TB drugs for 3 weeks | INH, RFT, EMB, LFX |
| P3 | 46 | female | PTB | INH, RIF, EMB, PZA | Stopped all anti-TB drugs for 2 weeks | INH, RFT, EMB |
| P4 | 50 | male | EPTB | INH, RIF, EMB, PZA | Stopped all anti-TB drugs for 2 weeks | INH, RFT, EMB, LFX |
| P5 | 37 | female | EPTB | INH, RFT, EMB, PZA | Stopped all anti-TB drugs for 2 weeks | INH, RFT, EMB |
| P6 | 32 | male | PTB | INH, RFT, EMB, PZA | Stopped PZA | INH, RFT, EMB |
| P7 | 54 | female | PTB | INH, RIF, EMB, PZA | Stopped all anti-TB drugs for 2 weeks | INH, RFT, EMB |
| P8 | 30 | male | PTB | INH, RFT, EMB, PZA | Stopped PZA | INH, RFT, EMB |
| P9 | 19 | female | PTB, EPTB | INH, RIF, EMB, PZA | Stopped PZA | INH, RFT, EMB |
| P10 | 29 | female | PTB | INH, RIF, EMB, PZA | Stopped PZA | INH, RFP, EMB |
| P11 | 43 | female | PTB | INH, RIF, EMB, PZA | Stopped PZA | INH, RFP, EMB |
| P12 | 26 | female | PTB | INH, RFT, EMB, PZA | Stopped PZA | INH, RFP, EMB |
| P13 | 48 | female | EPTB | INH, RIF, EMB, PZA | Stopped PZA、RIF | INH, EMB |
| P14 | 47 | male | PTB | INH, RIF, EMB, PZA for the first two months and PTO, MFX, RFT, PZA for the next two months | Stopped all anti-TB drugs for 2 weeks | PTO, MFX, RFT，LZD |

PTB: pulmonary tuberculosis, EPTB: extrapulmonary tuberculosis, INH: isoniazid, RIF: rifampin, RFT: rifapentine, EMB: ethambutol, PZA: pyrazinamide, LFX: levofloxacin, MFX: moxifloxacin, PTO: prothionamide, LZD: linezolid.

**Supplementary Table8 Sample compositions of the discovery cohort**

| **group** | **T1** | **T2** | **T3** | **T4** | **T5** |
| --- | --- | --- | --- | --- | --- |
| ATB-DILI group (n=9) | 9 | 9 | 9 | 9 | 8 |
| ATB-Ctrl group (n=9) | 9 | 9 | 9 | 9 | 9 |
| HC group (n=18) | 18 | - | - | - | - |

ATB-DILI group: T1: baseline, T2: one-third course, T3: two-third course, T4: timepoint of ATB-DILI occurrence, T5: recovery period.

ATB-Ctrl group: T1: baseline, T2: 2W after medication, T3: 4W after medication, T4: 6W after medication, T5: 8W after medication.

**Supplementary Table9 Fold change and p value of candidate biomarkers**

| **Candidate biomarkers** | | **ATB-DILI T4/T1** | |  | **ATB-DILI T4/**  **ATB-Ctrl T4** | |  | **ATB-DILI T4/HC** | |
| --- | --- | --- | --- | --- | --- | --- | --- | --- | --- |
|  |  | FC | *p* |  | FC | *p* |  | FC | *p* |
| Bile acids | THCA | 20.73 | 4.36E-10 |  | 3.7 | 0.0102 |  | 27.75 | 1.30E-07 |
|  | GHCA | 15.17 | 1.81E-12 |  | 2.76 | 0.0029 |  | 18.68 | 1.08E-11 |
|  | TCA | 2.79 | 0.0006 |  | 2.81 | 0.0085 |  | 19.67 | 8.55E-05 |
|  | GCA | 2.07 | 0.0405 |  | 1.96 | 0.0045 |  | 7.55 | 0.0005 |
|  | TCDCA | 2.17 | 0.0008 |  | 1.99 | 0.0147 |  | 7.27 | 2.11E-07 |
|  | GCDCA | 1.87 | 0.0317 |  | 1.59 | 0.014 |  | 3.06 | 0.0001 |
| Fatty acids | MA | 1.97 | 0.0009 |  | 1.65 | 0.0489 |  | 1.84 | 0.0228 |
|  | POA | 2.6 | 0.0004 |  | 1.82 | 0.0673 |  | 2.26 | 0.0044 |
|  | LA | 2.06 | 0.0005 |  | 1.63 | 0.0963 |  | 2.02 | 0.0053 |
|  | ARA | 1.76 | 0.001 |  | 1.61 | 0.0674 |  | 1.72 | 0.0149 |
|  | OA | 2.07 | 0.0005 |  | 1.53 | 0.1275 |  | 2.1 | 0.0022 |
